# Exposure to Perfluorinated Alkyl Substances and Health Outcomes in Children: A Systematic Review of the Epidemiologic Literature

**DOI:** 10.3390/ijerph14070691

**Published:** 2017-06-27

**Authors:** Kristen M. Rappazzo, Evan Coffman, Erin P. Hines

**Affiliations:** 1 Oak Ridge Institute for Science and Education at the U.S. Environmental Protection Agency, National Center for Environmental Assessment, U.S. Environmental Protection Agency, Research Triangle Park, NC 27709, USA; rappazzo.kristen@epa.gov (K.M.R.); coffman.evan@epa.gov (E.C.); 2 National Health and Environmental Effects Research Laboratory, U.S. Environmental Protection Agency, Chapel Hill, NC 27709, USA; 3 Office of Air Quality Planning and Standards, Office of Air and Radiation, U.S. Environmental Protection Agency, Research Triangle Park, NC 27709, USA; 4 National Center for Environmental Assessment, Office of Research and Development, U.S. Environmental Protection Agency, Research Triangle Park, NC 27709, USA

**Keywords:** children’s health, perfluorooctane sulfonate (PFOS), perfluorooctanoate (PFOA), puberty, immunity

## Abstract

Perfluoroalkyl substances (PFAS), chemicals used to make products stain and stick resistant, have been linked to health effects in adults and adverse birth outcomes. A growing body of literature also addresses health effects in children exposed to PFAS. This review summarizes the epidemiologic evidence for relationships between prenatal and/or childhood exposure to PFAS and health outcomes in children as well as to provide a risk of bias analysis of the literature. A systematic review was performed by searching PubMed for studies on PFAS and child health outcomes. We identified 64 studies for inclusion and performed risk of bias analysis on those studies. We determined that risk of bias across studies was low to moderate. Six categories of health outcomes emerged. These were: immunity/infection/asthma, cardio-metabolic, neurodevelopmental/attention, thyroid, renal, and puberty onset. While there are a limited number of studies for any one particular health outcome, there is evidence for positive associations between PFAS and dyslipidemia, immunity (including vaccine response and asthma), renal function, and age at menarche. One finding of note is that while PFASs are mixtures of multiple compounds few studies examine them as such, therefore the role of these compounds as complex mixtures remains largely unknown.

## 1. Introduction

Perfluoroalkyl substances (PFAS) are highly stable carbon fluorine compounds that have been used since the 1940s as surfactants in products for stain-proof and grease-proof applications. Through biomonitoring the US population, it is known that certain PFAS are ubiquitous in the US population [1]. These compounds include perfluorooctanoate (PFOA), perfluorooctane sulfonate (PFOS), and perfluorononanoic acid (PFNA), among others. Primary exposure routes to PFAS include food and water [2]. Like many other environmental compounds, it has been demonstrated that children have a higher body burden, i.e., elevated serum concentrations of a PFAS, versus adults [1,3]. This is thought to be due to mouthing behaviors, time spent on the floor closer to dust sources, different body size to surface area ratios across ages, exposure through placental transfer, or breastfeeding [4]. The increased burden of PFAS in children has the potential to affect health throughout childhood, development and potentially later in life. The current and future health of a child can be affected by his/her physical environment, in utero experience, nutrition, socio-economic status, educational level, and the built environment [5,6,7,8,9]. It is increasingly understood that exposure to environmental chemicals during sensitive windows has the potential to permanently alter a child’s risk of future morbidity, even at doses that have little effect in adults.

Multiple potential mechanisms of action exist for PFAS. Because PFOA and PFOS have been studied most extensively in laboratory animals, most is known about these PFAS. Of special interest to this review are mechanisms that may affect the developing organism. Potential mechanisms of action include peroxisome proliferation [10], mitochondrial dysfunction and oxidative stress [11,12], effects on reproductive hormones including progesterone [HE1] [13,14], estrogen [14], inhibin [13], and testosterone [15], loss of gap junction intercellular communication [16], and interruption of thyroid function [17]. In the liver, peroxisome proliferation is much stronger in laboratory rodents than in humans [10], likely contributing to inter-species cholesterol differences with PFCs including hypolipidemia or decreased cholesterol in mice and hyperlipidemia in humans. Thus many potential mechanisms could contribute to PFAS-dependent effects on children’s health.

Environmental chemicals like PFAS are often studied as individual compounds but in fact exist as complex mixtures in the environment. These mixtures can change over time as older chemistries are phased out and replacement chemistries are introduced. Legacy chemicals with long half-lives can be persistent in the environment and humans long after they are phased out. Many perfluorinated chemicals have been measured in human biological samples, some of which are found ubiquitously in serum of greater than 99% of people sampled in national surveys. Mechanisms of action may be similar or disparate for chemicals within a PFAS mixture, may affect similar signaling pathways or may disrupt developing tissues or metabolic pathways. Many of the PFAS exist in humans and the environment as mixtures, but little is known of the role of these compounds as complex mixtures.

The purpose of this review is to capture and characterize peer-reviewed epidemiologic literature examining associations between PFAS in biological media from children or pregnant women and various health outcomes in children.

## 2. Methods

A systematic review of the literature was performed by searching PubMed for studies on perfluorinated compounds and child health outcomes; returned references had to include both a PFAS term and a child health outcome term. Search terms included an extensive list of perfluorinated compounds and health/exposure terms ranging from general (e.g., child or children) to specific (e.g., Attention Deficit Hyperactivity Disorder (ADHD)) and were based on authors’ knowledge of topic and key terms from known research (see Supplementary Materials Table S1 for search term string). Searches were performed by joining two parenthetical terms with an AND operator. The first term was comprised of PFAS terms linked with OR operators, and the second with health/other terms linked with OR operators. We conducted the search in two forms: (1) “simple” wherein PFAS terms were limited to PFOA, PFOS, PFAS, and PFC and full where all PFAS terms (Table S1) were used. Duplicates were removed from results. Limits applied to the search included human subjects and English language. The initial search returned over 1953 articles that were screened by authors for inclusion first based on title and abstract. Of these, 42 articles were retained for further screening based on the full article, and 38 were included in the final review. The search was updated through June 05, 2017 and 27 new studies were added, and one study was removed due to academic dishonesty concerns (the supervising author Anoop Shankar was discovered in 2012 to have falsified his resume, regarding his degrees and publication history. The removed study was accepted before this occurred and was in an area with few other studies (kidney function). He is also an author on two other papers that we decided to include in this review as they were published in 2014 and in outcomes that had a more substantial body of work (cardiometabolic)), for a total of 64 included studies. For inclusion in this review we required serum, blood, or breast milk concentrations of PFAS that were measured concomitantly with the health outcome (e.g., serum PFAS and triglyceride concentrations) or early in life and associated with a later health outcome (e.g., PFAS in cord blood and behavioral outcomes in children). Studies were also required to report effect estimates in children under 18 years of age (selected effect estimates are presented in Supplementary Materials Table S1). Because there have been several recent reviews of birth outcomes, specifically of fetal growth, in association with PFAS, birth outcomes were not included as part of this review [18,19,20].

A risk of bias analysis was performed to evaluate the methodological design and implementation of the studies included in our review (see Supplementary Materials, PFAS Children’s Health Risk of Bias Criteria). Risk of bias criteria were derived and adapted from a systematic review of PFOA effects on fetal growth [21], which developed its risk of bias framework from the Cochrane Collaboration’s Risk of bias tool and the Agency for Healthcare Research and Quality’s criteria [22,23]. Seven criteria for risk of bias were considered: selection bias, exposure assessment, outcome assessment, confounding, missing data, conflict of interest, and “other”. The studies included in our review were assigned a risk of bias score for each of the seven categories of interest. Risk of bias scores, determined by a single reviewer (Evan Coffman) according to the set of guidelines for each category, were assigned as “low risk”, “probably low risk”, “moderate or unclear risk”, “probably high risk”, or “high risk”. The assigned scores and justification of the scores were evaluated by the other two reviewers (Kristen M. Rappazzo and Erin P. Hines), and any disagreements were resolved by selecting the most conservative judgment, as outlined by Johnson, Sutton, Atchley, Koustas, Lam, Sen, Robinson, Axelrad and Woodruff [19].

## 3. Results

All included studies are summarized in Supplementary Materials Tables S2–S7. Study designs of included articles are primarily cohort or cross-sectional. Study populations tended to be of the general population, including several longitudinal cohort studies such as the Danish National Birth Cohort—a large cohort of pregnant women established to examine exposures during pregnancy and early life, and health outcomes throughout life [24]. Alternatively, study populations also included those with known increased exposure to PFAS, such as the C8 cohort, a population from the mid-Ohio valley with documented elevated body burdens of PFOA due to living near point sources of known PFAS release into the environment [25]. Several population sources are examined repeatedly in the studies included in this review. These include the C8 cohort (n = 8) and the Danish National Birth Cohort (n = 7), as well as the United States National Health and Nutrition Survey (NHANES, n = 8), among others. PFAS concentrations had considerable variability across the study, with mean values ranging from 0.57 to 111 ng/mL across studies for PFOA and PFOS. Measurement of PFAS occurred primarily in serum (including maternal serum) rather than blood, and only a single study used breast milk as a medium. Studies occurred predominantly in the United States (US), Taiwan, or Scandinavian countries, and covered periods from the late 1970s through the early 2010s. Outcomes covered by the included studies include: neurodevelopment and attention (n = 19), cardio-metabolic measures (n = 16), asthma, infection, and immunity (n = 13), pubertal onset indicators (n = 6), thyroid function (n = 7), and renal function (n = 4).

### 3.1. Neurodevelopment and Attention

The brain and nervous system development is a dynamic process, occurring over various lifestages with important programming and vulnerability at each stage of development [26,27]. Alterations in neurodevelopment have the potential for life-long impact on quality of life. Multiple studies have examined associations between PFAS and neurological outcomes in children, including effects on attention, behavior, motor activity, learning, cognition, and development. Summaries of studies in neurodevelopment and attention are presented in Supplementary Materials Table S2.

Studies that examined childhood developmental milestones or neurodevelopment report primarily null results, though some observed positive associations. In the Danish National Birth Cohort, prenatal PFOA or PFOS concentrations (measured in plasma from maternal blood taken during the 1st trimester) were not associated with changes in timing to developmental milestones in infants including measures of fine motor control, attention, cognition, and language at 18 months of age [28]. One measure of gross motor control (sitting without support) had inverse hazard ratios with increasing PFOS exposures, and to a lesser degree with PFOA [28]. In an examination of the same cohort at age 7, no associations were found between prenatal PFOS or PFOA and behavioral or motor/coordination problems [29]. In a cohort of newborns, Donauer, et al. [30] observed increased odds ratios (OR) for hypotonicity (low muscle tone, aka “floppy baby syndrome”) with log increases in PFOA (maternal serum), though confidence intervals were not reported. Neither PFOA nor PFOS had associations with attention in this cohort [30]. In children from the Taiwan Birth Panel Study, cord blood PFAS were assessed for association with neurodevelopment in 2 year-old children using the Comprehensive Developmental Inventory for Infants and Toddlers, a test with subdomains of cognitive, language, motor, social and self-help; both change in score and odds of poor performance were examined [31]. Chen et al. (2013) [31] observed no association with PFOA and the neurodevelopmental markers examined, but positive ORs of poor performance were observed with increasing PFOS, though linear score changes were less pronounced. In contrast, a Norwegian study that also examined both cognitive and psychomotor development scores and behavioral problems reported generally null associations for PFOA or PFOS with outcomes; though PFAS were measured in breast milk rather than cord blood [32]. Goudarzi, et al. [33] used the Hokkaido Study on Environmental and Children’s Health to examine prenatal PFAS in maternal serum and indices of psychomotor or mental development at 6 and 18 months, finding no evidence of associations. They did observe a negative association with PFOA levels and the mental development index in girls at 6 months, but it did not persist at 18 months [33]. In the Ohio Valley C8 cohort, neither PFOA measured in utero or in childhood (serum) was associated with any IQ measure (full, verbal, or performance), academic measure, neuropsychological function score, or attention measure [34]. Likewise, Wang, et al. [35] generally found null results for maternal serum PFAS, including PFOA and PFOS and IQ in the Taiwan Maternal and Infant Cohort Study. In the C8 study, Stein and Savitz [36] found inverse ORs of learning problems with increasing levels of serum PFOS and PFNA, and potentially inverse ORs with PFOA. In another Ohio based cohort, the Health Outcomes and Measures of the Environment (HOME) Study, natural log increases in maternal serum PFOS were associated with increased odds of poorer behavioral regulation, metacognition, and global executive function, while other PFAS were not [37]. In a Danish cohort, Strom, et al. [38] observed no associations between scholastic achievement and maternal serum PFAS.

Nine studies examined either ADHD or related indicators of impulsivity; in general, results for these studies are mixed. In a cross-sectional U.S. study, Gump, et al. [39] found lower response inhibition/impulsivity was associated with increasing PFAS exposures (blood); associations with PFOA were of lower magnitude than associations with other PFAS. One cross-sectional study of NHANES data observed increased odds of parent-reported ADHD with increased serum PFOA, PFOS, and perfluorohexane sulfonate (PFHxS) [40]. Stein and Savitz [36] reported positive associations in the C8 cohort between PFOS and PFHxS, but not PFOA or PFNA, and ADHD after adjusting for medication use. In a cohort across Greenland, the Ukraine, and Poland, Hoyer, et al. [41] observed log-increases in maternal serum PFOA to be associated with increases in both hyperactivity and behavioral problems, and log-increases in maternal PFOS to be associated with hyperactivity. Liew, et al. [42] reported potential positive associations between ADHD and maternal plasma PFOA in the Danish National Birth Cohort, though some associations were only observed when examining quartiles of exposure. In a cohort created by combining data from the Taiwan birth panel study and the Taiwan early-life cohort, Lien, et al. [43] found cord blood PFNA concentrations to be associated with inattention, impulsivity/hyperactivity, and oppositional defiant disorder as measured by the Swanson, Nolan, and Pelham IV scale but not neurobehavioral symptoms measured by the Child Behavior Checklist (CBC) or the Strengths and Difficulties Questionnaire. They also found only null associations between PFOA, PFOS, and PFNA and neurobehavioral symptoms of ADHD [43]. Quaak, et al. [44], using the Dutch Linking Maternal Nutrition to Child Health cohort (PFAS measured in cord blood), found higher PFOA tertiles associated with potential decreases in externalizing behavior using the CBC, but no associations with the ADHD scale, and no associations with PFOS concentrations; however, they did observe potential negative associations between summed PFAS and both externalizing behavior and ADHD. Other studies have also observed null or negative associations. Ode, et al. [45] reported null ORs between cord serum PFAS and ADHD in a case-control study of Swedish children. In a Danish cohort, Strom, Hansen, Olsen, Haug, Rantakokko, Kiviranta and Halldorsson [38] observed inverse ORs for ADHD with maternal serum PFOA and PFOS, though confidence intervals were large. A study of C8 children examined serum PFOA with mother and teacher reports of executive function, ADHD like behavior, and behavioral problems using standardized score metrics [46]. This study found that associations depended on who was reporting and that associations differed by child’s sex. If mothers were reporting, boys had lower scores (indicated fewer behavioral issues) and girls had higher scores with doubling of PFOA concentrations. If teachers were reporting, boys still had lower scores with increasing PFOA, but no associations were observed in girls [46].

One study also examined autism in a case-control study from the Danish National Birth cohort, and found no association with maternal plasma PFOA, PFNA, perfluoroheptanesulfonate (PFHpS), or perfluorodecanoic acid (PFDA) concentrations [42]. PFOS and PFHxS had elevated ORs, although PFOS confidence intervals were wide [42].

Effects for observed neurological outcomes across studies are inconsistent; while some studies observe positive associations for both ADHD and neurodevelopment, there are also several studies that observe negative and null associations. Recent reviews on prenatal exposures to environmental chemicals in association with children’s neurodevelopment report similar findings [47,48]. There is some evidence of neurodevelopmental impairments in animals exposed to PFAS. Johansson, et al. [49] demonstrated neurobehavioral deficits in adult mice after neonatal PFOA and PFOS exposure, where spontaneous behavior of adult mice manifested as hyperactivity and inability to habituate. In other toxicological models, fish showed early attainment of habituation with PFOS exposure but elevated spontaneous activity; mechanistic understanding of these changes via pharmaceutical manipulation revealed that multiple neurotransmitter systems including norepinephrine, serotonin, and dopamine were involved in these changes [50]. Also in fish, chronic PFOS-dependent behavioral effects were lifestage specific and spilled over into the next generation [51]. Despite the evidence from animal literature, the mixed nature of findings in humans precludes firm conclusions for the effects of PFAS on neurological outcomes.

### 3.2. Cardiometabolic

A number of cardiometabolic outcomes, a clustering of conditions that increase the risk for coronary artery disease, stroke, and type 2 diabetes, have been studied in association with PFAS exposure, including overweight or obesity status; factors relating to glucose regulation and metabolic syndrome; and measures of dyslipidemia, abnormal levels of serum total cholesterol, low-density lipoprotein cholesterol (LDL-C), high density lipoprotein-cholesterol (HDL-C), or triglycerides. Overweight or obesity status is itself associated with many poor health outcomes, and puts a child at greater risk for adult morbidity. Disruption of glucose regulation may lead to serious conditions such as Type 2 diabetes. Dyslipidemia in children may lead to earlier development of atherosclerosis and cardiovascular disease [52]. Summaries of studies of cardiometabolic outcomes are presented in Supplemental Materials Table S3.

Nine studies examined anthropometric outcomes, such as weight; of these, eight have done so using prenatal or maternal PFAS concentrations. In studies examining prenatal PFAS concentrations and anthropometric measures of children in the Danish National Birth Cohort (plasma), Andersen, et al. [53] observed null associations between weight, height, or BMI at 5 or 12 months of age with PFOA or PFOS. In a follow up study with the children at 7 years of age, BMI and waist circumference continued to have no associations with PFOA or PFOS [54]. However, overweight status had inverse ORs, with wide CIs, with increasing quartiles of PFOS [54]. In the Ohio based HOME study, maternal serum PFOA concentrations were associated with higher risk of overweight/obesity at 8 years of age, and with BMI z-score, waist circumference, and body fat percentage; these associations were non-linear, with the 2nd tertile effect estimate being higher than the 3rd [55]. In Bristol England, the Avon Longitudinal Study of Parents and Children (ALSPAC) examination of prenatal PFAS (maternal serum) and weight in girls at 20 months of agefound an increase in weight of 580 g (301, 858 g) when adjusting for birthweight and height at 20 months for the highest tertile ofPFOS compared to the lowest; they observed no associations between PFOA or PFHxS and weight at 20 months in girls, Maisonet, et al. [56]. Alternately, a study examining the children from the Danish Pregnancy Cohort, with a mean age at follow-up of 20 years, observed positive ORs (BMI and waist circumference) with increasing prenatal PFOA exposure (serum) in female offspring but null associations in males and in association with prenatal PFOS [57]. In a cohort from Greenland and the Ukraine, Hoyer, et al. [58] observed only slightly elevated ORs with maternal plasma PFOA and no associations with PFOS for overweight status, but both were associated with increased odds of waist-to-hip ratio >0.5 at five to nine years of age. Height z-score, but not weight, was negatively associated with 3rd trimester maternal serum concentrations of several PFAS (excepting PFOA) for children aged 2 to 11, particularly in girls, in the Taiwan Maternal and Infant Cohort study [59]. In a cross-sectional study, Timmermann, et al. [60] found no associations between serum PFOA or PFOS at age 8 years and anthropometric measures, including BMI. A Danish pregnancy cohort from 1988 to 1989 reported BMI significantly increased across tertile of maternal serum PFOA measured at gestational week 30 in female offspring at age 20, though no effect estimates were reported and interquartile ranges were within normal values [61].

Five cross-sectional studies examined dyslipidemia. In adolescents from NHANES, increases in PFOA, PFOS, or total PFAS serum concentrations were positively associated with high total cholesterol (>170 mg/dL) and high LDL-C; PFAS were not associated with clinically abnormal HDL-C and triglyceride levels [62]. Linear associations were similar, with increases in PFAS associated with increases in total cholesterol and LDL-C, and no associations with triglycerides or HDL-C, though HDL-C levels did show small decreases with increases in plasma PFOA levels [62]. Also in NHANES, Lin et al. [63] found no associations between PFASs and HDL-C or triglyceride levels examined as components of metabolic syndrome in NHANES adolescents. Children and adolescents from the Ohio Valley C8 population had PFOS and PFOA serum concentration associated with increased odds of abnormal total cholesterol and LDL-C; PFOS was also associated with decreased odds of abnormal HDL-C [64]; Linear changes in lipids were also examined, with [64] reporting positive though non-linear trends between PFOS and total cholesterol, LDL-C, and HDL-C. A Danish study found no associations between serum PFOA or PFOS and triglycerides in normal weight children, but did find increases in PFOA or PFOS associated with increases in triglycerides in overweight children [60]. A study in Taiwan found log increases in serum PFOA, PFOS, and PFNA associated with increases in total cholesterol, LDL-C, and triglyceride concentrations [65]. In the ALSPAC cohort examination of lipids in association with maternal serum PFAS, Maisonet, et al. [66] observed positive, though non-linear and non-monotonic, associations with increases in maternal serum PFOA and PFOS and total and LDL-cholesterol.

In a cross-sectional analysis of metabolic syndrome components and indicators among NHANES adolescents (12–17 years), Lin, Chen, Lin and Lin [63] observed a positive association between doubling of serum PFNA concentrations and the glucose component of metabolic syndrome, defined as glucose levels ≥5.55 mmol/L or a self-reported use of anti-hyperglycemic medications (OR: 3.15 (1.39–7.12)); however, other metabolic components and overall metabolic syndrome had inverse or null ORs with PFNA. PFNA was also associated with decrements in measures of insulin resistance and B cell function [63]. Other PFAS (PFOA, PFOS, and perfluorohexanesulfonic acid (PFHS) had null or negative associations with metabolic syndrome components and primarily null associations between biomarker measures [63]. In a small Taiwanese cohort, metabolic indicators (insulin, glucose, etc.) were not associated with increasing serum concentrations of PFOA, PFOS, PFNA, or perfluoroundecanoic acid (PFUA) [67]. A cross-sectional study in Denmark observed no associations between serum PFOA or PFOS and percent change in insulin concentrations in normal weight children, but among overweight children both PFOA and PFOS were associated with increased insulin concentration, higher B-cell activity, and elevated insulin resistance [60]. In the 20 years follow-up of the Danish Pregnancy Cohort, there were positive associations with percent changes of insulin and leptin association and log-unit increases in prenatal serum PFOS [57]. Studies also examined adiponectin, a hormone that plays a role in glucose regulation and fatty acid oxidation, and is important for metabolic homeostasis. In a cohort of hypertensive young people from Taiwan (aged 12–30), higher PFNA serum concentration was associated with elevated serum adiponectin concentration, while PFOA, PFOS, and PFUA were not [67]. Although in this study only the *p*-value for trend and mean response values were reported, rather than comparison effect estimates and the population included adults. In the Danish Pregnancy Cohort, prenatal PFOA exposure (serum) was negatively associated with adiponectin concentrations [57]. Both the study of the Danish Pregnancy Cohort and a cross-sectional study in Denmark observed no associations between PFOS and adiponectin [57,60].

A single study of NHANES examined blood pressure and hypertension in children, finding no associations between blood pressure measurements and increases in serum PFOA or PFOS [52]. Another cross-sectional study examined carotid artery intima-media thickness in a Taiwanese population of which 38% had elevated blood pressure during childhood, finding increased carotid artery intima-media thickness in adolescents aged 12–19 with increasing quartiles of plasma PFOS [68].

Carido-metabolic effects in children were reported in multiple studies. Analyses reported generally higher or abnormal levels of total cholesterol and LDL-C in association with PFAS serum concentration. Some mechanistic analyses have also been performed by Fletcher et al. (2013) [69], who found changes in the expression of genes involved in cholesterol metabolism (transport and mobilization) to be associated with serum PFAS in the C8 population. The evidence for effects on weight or BMI in children across PFAS is mixed with PFOA most frequently associated with overweight status in females but some PFOA studies also show null results. It may be that small positive associations, such as those reported by Maisonet, Terrell, McGeehin, Christensen, Holmes, Calafat and Marcus [56], have cumulative effects over time, which lead to being overweight in adulthood. Rodent models of glycemic control with perinatal exposure to PFAS affected glucose tolerance in adult offspring, including enhanced effects on a high-fat diet [70], and altered signaling pathways like leptin [71]. It is important to note that most of the studies reviewed here are cross-sectional, so temporality cannot be established, and do not account for dietary intake, which can be an import source of PFAS [72] and therefore a potential confounder. Studies of glucose regulation in children have generally reported mixed effects, with limited agreement between studies. A single study of carotid artery intima-media thickness in adolescents found an association with PFOS concentration. The strongest evidence for a relationship between PFAS exposure and cardiometabolic effects in children comes from studies of dyslipidemia.

### 3.3. Immunity, Allergic Response, Infection, and Asthma

Children are protected from infectious agents by innate and adaptive immunity, which is increasingly understood to be modifiable by our environment. Allergic and immunological responses can also be modulated by exposure to exogenous environmental compounds. Vaccinations substantially reduce disease, disability, death and inequity worldwide [73]. Recent work shows that inoculation with the measles vaccination is associated with an elevated level of protection against other infectious diseases [74]. Because the efficacy of a vaccine depends on the quality of the immune response, it is important to understand if environmental exposures may modulate this response. Summaries of studies in immunity, allergic response, infection, and asthma are presented in Supplementary Materials Table S4.

Three studies examined suppression of vaccine-mediated antibody response in association with PFAS. In a Faroe Islands birth cohort, Grandjean, et al. [75] examined serum PFAS concentrations prenatally and at age 5 in association with tetanus and diphtheria serum antibody titers at age 5 (prior to vaccination booster) and at age 7 (after booster). Antibody concentrations at age 5 were generally not associated with combined PFAS concentrations, except diphtheria where a doubling of prenatal PFAS concentration was associated with a substantial decrease in antibody concentrations [75]. PFAS concentrations at age 5, including when adjusting for prenatal PFAS, have strong negative associations with antibody concentrations for both tetanus and diphtheria at age 7 [75]. For individual PFAS, associations with antibody concentrations at age 7 are congruent with the results for total PFCs [75]. However, prenatal PFOS showed a strong negative association with diphtheria antibody concentration at age 5; prenatal PFNA and PFDA also had negative associations with diphtheria antibody concentrations at age 5. Grandjean, Andersen, Budtz-Jorgensen, Nielsen, Molbak, Weihe and Heilmann [75] also examined odds of antibody levels falling below a clinically protective level (0.1 IU/mL), observing positive ORs for diphtheria and tetanus at age 7 with a two-fold increase in PFOS at age 5; results were similar for PFOA. People from the Faroe Islands have higher persistent organic pollutant (i.e., PCB) and methylmercury serum concentrations than those from the general US population [76]; in this study, PFAS and PCB concentrations were not correlated with each other, and adjustment for PCBs in this model did not appreciably change the results [75]. Granum, et al. [77] also examined antibody concentrations in a subcohort of the Norwegian Mother and Child Cohort Study (MoBa) study. Increases in maternal plasma PFAS concentrations at delivery (PFOA, PFOS, PFNA, PFHxS) were negatively associated with children’s anti-rubella antibody titer at three years of age [77]. They also observed potential associations between PFOS and PFOA and measles vaccine antibody concentrations, however these associations were unadjusted for potential confounders [77]. In NHANES (1999–2000 and 2003–2004), Stein et al., (2016) [78] found serum PFOA and PFOS to be associated with decreases in rubella and mumps antibodies in children 12–19 years old. These studies show some effect of PFAS serum concentration on suppression of antibody response to vaccination.

Six recent studies have examined asthma in association with PFAS. In the Taiwanese Genetics and Biomarkers study for Childhood Asthma, Dong, et al. [79] found positive ORs and increasing trends for asthma with serum PFOA, PFOS, PFDA, PFHxS, and PFNA. Perfluorobutanesulfonic acid (PFBS) and perfluorododecanoic acid (PFDoA) had positive ORs for asthma, though without a clear trend or only at the highest exposure levels [79]. In this study, asthmatic children were recruited from hospitals, while non-asthmatic children were recruited from schools. If the school population was not similar to the population that gave rise to the asthmatic population bias may have been introduced [79]. In a subset of the same population, Qin et al., [80] observed decrements in metrics of lung function (forced expiratory volume, forced expiratory flow 25–75%, and forced vital capacity) with doubling of several PFAS concentrations (PFOA, PFOS, PFHxS, PFNA) in children with asthma but not in children without asthma. In a cross-sectional study using adolescent NHANES participants, Humblet, et al. [81] observed a positive OR between self-report of ever having asthma and increasing serum PFOA concentration; PFNA also had a positive OR but a wide CI. Generally null associations were observed for other PFAS (PFOS and PFHxS) and ever asthma, or for any PFAS and current asthma or wheeze [81]. In a subset of that population (NHANES 2005–2006) Stein et al., [78] found similar elevated ORs with wide CIs for PFOA, PFNA and asthma; they also reported elevated ORS with PFOS, but not PFHxS. In another cross-sectional analysis of asthmatic and non-asthmatic children in Taiwan, increasing quartiles of serum PFOA were associated with increasing odds of asthma [82]. PFOS, PFBS, PFDA, PFHxS, and PFNA were also associated with asthma, for some PFAS the associations were divergent by sex (PFOS only associated with asthma in males) or potentially sex divergent (PFBS had stronger effect in males), while the others had similar effects across sexes [82]. In a cohort across Greenland and the Ukraine Smit, et al. [83] reported generally null associations between asthma or wheeze and a factor representing maternal plasma PFAS concentrations.

Five studies looked at allergies or similar outcomes, generally finding null results. In the two analyses of the Hokkaido Study on Environment and Children’s Health, no associations with maternal serum PFOA or PFOS and food allergies or eczema, and null to potentially negative associations with total allergies, were observed [84,85]. A cohort in Greenland and the Ukraine reported no association between a factor representing maternal plasma PFAS concentrations and current or ever eczema in children [83]. Wang et al. (2011) [86] observed no associations with increasing quartiles of cord blood serum PFOA and atopic dermatitis; they did report positive associations with PFOS and PFNA, though both had large confidence limit ratios, indicating low precision, and no trends. In a cross-sectional analysis of NHANES data, Stein et al., [78], observed associations with increased PFOA, PFOS and rhinitis; there was some evidence for associations with allergy as well, but no PFAS were associated with wheeze.

In addition to vaccination antibodies, Granum, Haug, Namork, Stolevik, Thomsen, Aaberge, van Loveren, Lovik and Nygaard [77] also examined allergen-specific IgE antibodies, using a test that distinguishes atopic and non-atopic status, reporting no associations between atopic status and concentrations of any PFAS (plasma) in the Norwegian cohort. Newborn immune function markers were examined in a cohort of 10 Canadian cities and generally null associations were observed between immune function and any maternal serum PFAS [87]. In a Japanese cohort, increasing maternal serum PFOA concentrations were negatively associated with cord blood IgE in 18 months old girls but not boys [85]. In the Taiwan Birth Panel cohort study, IgE levels at 2 years of age were not associated with PFOA, PFOS, or PFNA in cord blood serum [86]. Wang, Hsieh, Chen, Fletcher, Lien, Chiang, Chiang, Wu and Chen [86] also observed positive associations between PFOS and PFOA and IgE levels, both measured in cord blood, but only in boys. Another study in Taiwan examined IgE levels in children with and without asthma, reporting statistically significant p-values for trend with increasing concentrations of serum PFAS (PFOA, PFOS, PFDA, PFDoA, PFNA, and PFTA), though linear changes were not reported [79]. In a cross-sectional analysis of asthmatic boys, Zhu et al. [82] observed higher levels of IgE, and Th1 and TH2 cytokines, which might have contributed to asthma development, with higher levels of serum PFOS, PFOA, and PFDA.

Two studies examined susceptibility to infections or diseases. In the Danish National Birth Cohort, Fei, et al. [88] saw no associations between prenatal exposure to PFOA or PFOS (serum) and risk of hospitalizations for infectious disease in the first year of life. Granum, Haug, Namork, Stolevik, Thomsen, Aaberge, van Loveren, Lovik and Nygaard [77] observed positive associations between maternal plasma PFOA and PFNA and common cold incidence in the first 3 years of life and between PFOA and PFHxS and gastroenteritis. However, these associations were unadjusted for potential confounders [77].

Studies of individual health outcomes are limited in number, therefore conclusions should be made with caution; current evidence potentially suggests that antibody response to vaccination and asthma may be influenced by PFAS. The studies of vaccine response were well done cohort study designs and despite the small number offer compelling evidence. The asthma studies are less consistent and include a broader range of study designs and quality. There is no evidence for relationships between PFAS and IgE levels, allergy, and infection. In the one study that looked across these outcomes, several positive associations were observed and these in combination may indicate that prenatal PFAS exposure is linked to childhood humoral immunomodulation [77], which is supported by animal studies [89].

### 3.4. Pubertal Onset Indicators

Puberty is the process of sexual maturation, occurring over several years. Early onset or delayed onset puberty can be considered an important indicator of endocrine disruption, and has been associated with altered risk of adult disease: diabetes mellitus, heart disease, bone disease, substance abuse, and asthma [90,91,92,93].

Six studies examined pubertal onset indicators (see Supplementary Materials Table S5). Two used data from the ALSPAC cohort; one study focused on menarche before 11.5 years of age while the other examined hormone levels in a subset of girls. The other studies examined both age at menarche and other pubertal parameters, such as hormone levels, using case-control and cross-sectional designs [61,94,95,96]. Kristensen, Ramlau-Hansen, Ernst, Olsen, Bonde, Vested, Halldorsson, Becher, Haug and Toft [61] examined fetal exposure, through maternal serum at gestational week 30, to PFAS in a longitudinal cohort from Denmark, and observed a one-month delay in menarche per tertile increase of PFOA (or a 5.3 months delay from highest to lowest tertile of PFOA); no associations were observed with other reproductive parameters examined (e.g., cycle length, follicle stimulating hormone levels, etc.). Fetal PFOS levels were not associated with age at menarche [61]. A cross-sectional analysis of boys and girls in the C8 cohort found delayed menarche associated with both serum PFOA and PFOS concentrations, which held true after adjustment for the other PFAS [95]. Doubling of PFOS was associated with serum testosterone in males and estradiol in females indicating lack of sexual maturation (i.e., inverse odds ratios (OR) for levels above specified cut-offs), while increases in PFOA were not associated with estradiol or testosterone levels [95]. In a further examination of the C8 cohort, [97] found insulin-like growth factor, a marker of pubertal onset, to be negatively associated with serum PFOA (in girls), PFOS and PFNA (both boys and girls). It is important to note that menstrual blood loss is a potential route of PFAS excretion, thus in cross-sectional studies of girls with later pubertal onset may have higher PFAS levels than girls with earlier pubertal onset. Examining age at menarche from a different angle, Christensen, Maisonet, Rubin, Holmes, Calafat, Kato, Flanders, Heron, McGeehin and Marcus [94] used age at menarche before 11.5 years as their outcome in a case-control study of ALSPAC, finding null associations with most PFAS levels measured in maternal serum during pregnancy, but inverse ORs with PFOS. Inverse ORs may indicate the potential for delayed menarche with PFOS, or that girls with higher exposure to PFOS during fetal development were less likely to have early menarche. In a subset of the same population as Christensen, Maisonet, Rubin, Holmes, Calafat, Kato, Flanders, Heron, McGeehin and Marcus [94], higher levels of PFOA, PFOS, and PFHxS were associated with higher levels of total testosterone in girls, while no associations were observed with sex hormone-binding globulin concentrations [96]. A cross-sectional study in Taiwan examined follicle stimulating hormone levels in association with serum PFAS in 12–17 year olds, finding decreased FSH associated with increasing PFOS in boys and PFUA in girls; there was no evidence of associations for PFOA and PFNA [98].

The six studies of pubertal onset indicators have generally mixed results and varied study design. The most consistent evidence is for later age at menarche associated with either PFOA or PFOS exposure or both. This is supported by toxicological evidence from mouse models in which female offspring had delayed mammary gland development [99] and vaginal opening [100] with in utero and peri-pubertal exposure to PFOA, respectively.

### 3.5. Thyroid Function

Thyroid hormones are essential in regulating growth, development and metabolism and are especially vital for proper brain development early in life, with impairment known to affect neurological endpoints, including intelligence quotient (IQ). Five studies evaluated thyroid function in children in association with PFAS exposure (see Supplementary Materials Table S6).

In a cross-sectional analysis of the Ohio Valley C8 cohort children, Lopez-Espinosa, Mondal, Armstrong, Bloom and Fletcher [17] observed positive associations between clinical hypothyroidism and serum PFOA, while neither PFOS nor PFNA were associated with hypothyroidism. In children with no diagnosed thyroid disease, null associations were observed with PFOA and thyroid stimulating hormone (TSH) and total thyroxine (TT4) levels; TSH and TT4 levels were elevated with the highest concentrations of PFOS, and TT4 levels increased with increasing PFNA concentrations in participants without thyroid disease [17]. Lin, et al. [101] examined thyroid hormones and serum PFAS in a subset of a cohort of adolescents and young adults (12–30 year olds) from Taiwan with abnormal urinalysis (positive tests for any two of: proteinuria, glucosuria, or hematuria). Only PFNA was associated with free T4 levels, though the change was small (0.004 ng/dL (95%CI: 0.001–0.007)) per 1 ng/mL increase in PFNA) and was seen without a concomitant decrease in TSH that would be clinically expected [101]. No other PFAS were associated with thyroid hormone levels in this study. In a study of 52 boys and 31 girls in the Netherlands, increasing T4 levels in girls were associated with increasing cord blood PFOA concentrations [102]. No associations with T4 were observed with PFOS or with PFOA in boys [102]. In a cross-sectional analysis of the Taiwan Birth Panel study, doubling of cord blood plasma PFOS was associated with decreased T4 in boys, and increased TSH in both boys and girls though effects appeared to be non-linear and magnitude of effects was higher in boys [103]. PFOA, PFNA, PFUnDA were generally not associated with thyroid hormone concentrations [103]. A small South Korean study examined correlations between maternal PFAS during pregnancy and fetal T3, T4 and TSH measured in cord blood [104]. Fetal T3 was negatively correlated with PFOS and perfluorotridecanoic acid (PFTrDA), and had small negative correlations with PFOA and PFHxS [104]. Fetal TSH was positively correlated with PFOS and PFTrDA, while T4 was not substantially correlated with any PFAS [104]. In a Japanese study of 392 mother-infant pairs, maternal cord blood PFOS was positively associated with infant TSH levels, but not free thyroxine, and PFOA was not associated with either [105]. In another small South Korean study, infants with congenital hypothyroidism had higher mean serum levels of PFOA, PFNA, PFDA, PFUnDA, and total PFASs compared to healthy infants [106].

While some associations are observed between thyroid hormones and PFAS, no clear patterns emerge. There is some evidence for hypothyroidism, a finding that has also been observed in an adult NHANES population [107], but not in other studies of PFAS and thyroid function. Given the limited number of studies and the variability in the responses, no conclusions can be reached with certainty.

### 3.6. Renal Function

The function of the kidneys, or renal function, contributes to the normal homeostatic maintenance of blood pressure, removal of waste products from the body, red blood cell production and electrolyte balance. The long-term health risks of impaired kidney function can be substantial and can be exacerbated in children who are malnourished or those who are overweight or obese.

Four studies have investigated associations between PFAS and renal function in children (see Supplementary Materials Table S7). Two cross-sectional studies in the United States found associations between renal function and PFAS. Watkins, et al. [108] examined estimated glomerular filtration rate (eGFR) in association with serum concentrations of several PFAS, finding decrements in eGFR associated with increases in PFOA and PFOS concentrations and with log-increases in PFNA and PFHxS. Kataria, et al. [109], also using NHANES (2003–2010), found serum PFOA to be associated with eGFR and uric acid concentrations after adjustment for PFOS, while PFOS associations were attenuated after adjustment for PFOA. PFNA and PFHxS were not associated with eGFR or uric acid concentrations, and no PFAS were associated with serum creatinine concentrations [109]. Qin, et al. [110] examined 225 non-asthmatic children in Taiwan, finding serum concentrations of several PFASs associated with increased odds of high uric acid levels, including PFOA, PFOS, PFBS, PFDA, PFHxS, and PFNA. In a side analysis of a cohort of Taiwanese adolescents and young adults who had abnormal urinalysis, serum PFUA concentrations were higher in children with chronic renal failure [101], though only mean levels and *p*-values were reported, making quantitative interpretation difficult. While all studies of PFAS and kidney function in children to date have been cross-sectional, results from these studies provide evidence for interesting potential associations between PFAS and renal function in children using multiple different markers of kidney function.

### 3.7. Risk of Bias Analysis

**Figure 1:**
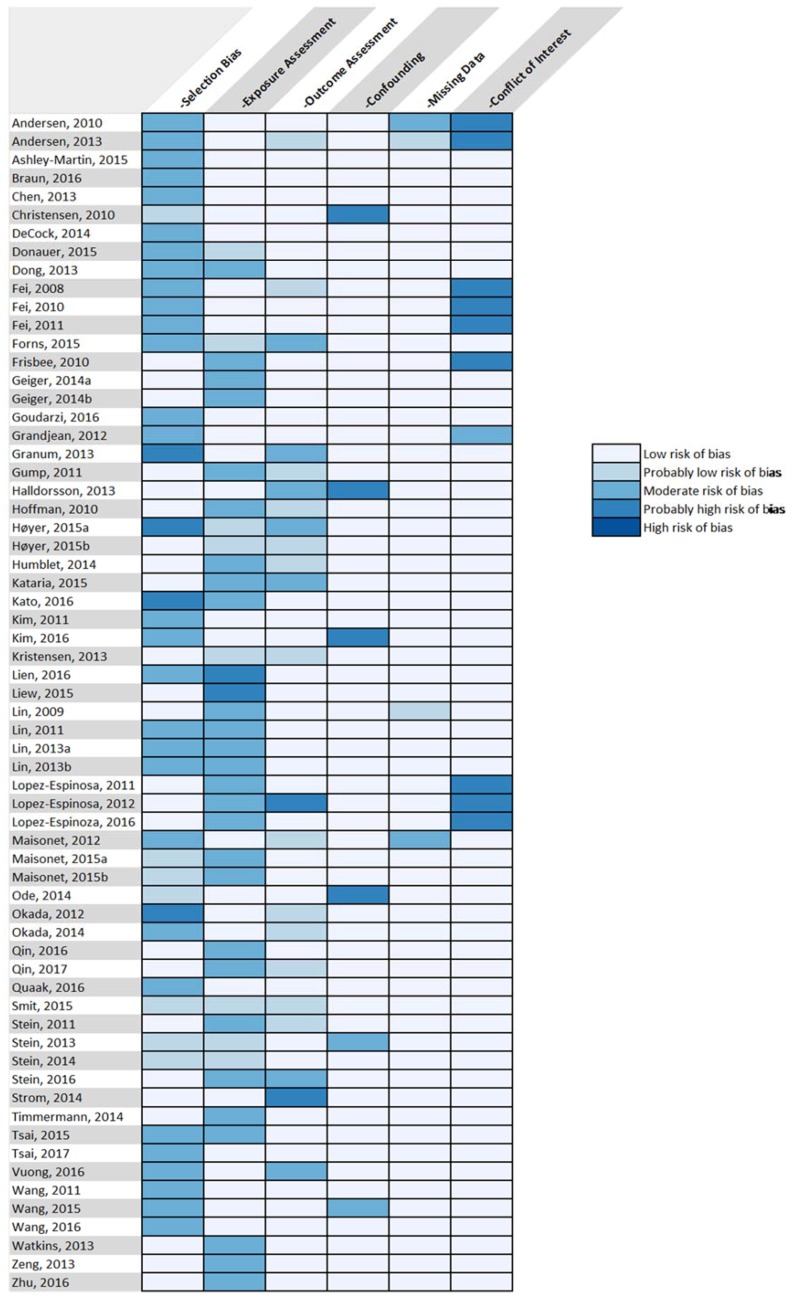
Classification of potential biases within each study.

**Figure 2:**
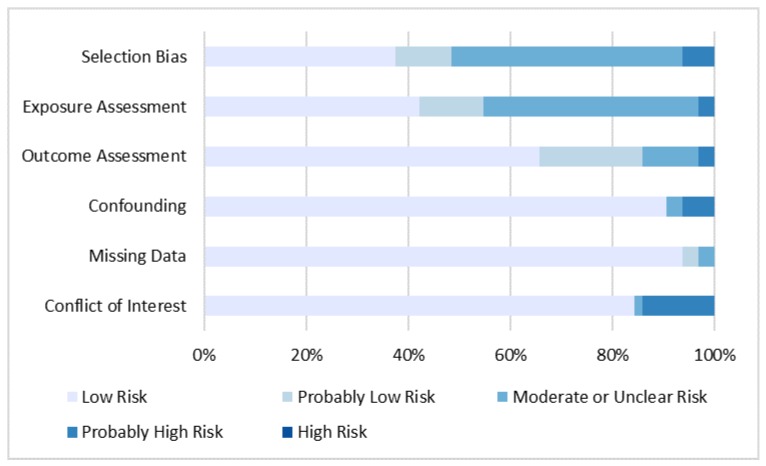
Distribution of potential sources of bias across included studies.

Based on the implementation of our risk of bias criteria, we determined there to be a low to moderate risk of bias in the studies included in our review. While all of the reviewed studies had potential biases relating to at least one of the seven categories of interest (Figure 1), the majority of these instances were classified as “moderate risk” or lower (Figure 2). Of the sources of bias considered, selection bias, exposure assessment, and conflict of interest were most commonly judged to present the highest risks. Participation and non-response bias were the greatest concerns within the selection bias domain. However, studies with high non-participation or non-response rates were determined to comprise lower risk if they had analyses demonstrating minimal differences between participants and non-participants. Although PFAS generally have long half-lives in humans [111], there is uncertainty regarding the use of concurrent PFAS levels as a surrogate for historical PFAS levels during the susceptible time range for the development of the outcome of interest. Due to the abundance of cross-sectional studies included in our review, many studies were found to have a moderate risk of exposure error, as timing of the exposure could not be ascertained.

## 4. Discussion

This systematic review summarizes childhood health outcomes associated with PFAS exposure from peer-reviewed studies that compare associations between measured concentrations in serum, cord blood, or breast milk in early life and a health outcome. Environmental chemicals like PFAS are often studied as individual compounds but exist as complex mixtures in the environment, and the role of these mixtures in children’s health is captured herein. The outcomes reported in the published literature with PFAS concentration measurements are diverse and include: cardiometabolic effects, anthropometric measurements, neurological, neurodevelopmental, and attention effects, asthma and immune response, thyroid changes, altered timing of puberty onset, and renal function. Physiological changes in childhood have the potential to alter adult disease risk and contribute to altered risk for adult morbidity and mortality. While there are a limited number of studies for any one particular health outcome, there is evidence for positive associations between PFAS and dyslipidemia, immunity (including vaccine response and asthma), renal function, and age at menarche.

The studies included in this review cover a number of health topics across childhood development. This gives a better, though not complete, picture of the potential for chronic health effects associated with PFAS exposure. These studies also cover both highly exposed populations, such as the C8 cohort, and populations from the general public. These populations may have discordant health outcomes due to differences in exposure or body burden of PFAS, and it is important to examine both types to best inform specific health outcomes. It also appears that some of the health associations observed in these studies show outcomes that differ by sex with only males or females as responders. Thus, it is important to examine effect measure modification on associations by sex when differential responses are suspected or expected. Overall the literature covers a substantial area of research and informs the knowledge of the overall potential impact of serum PFAS concentrations early in life on health outcomes.

Limitations of the studies reviewed here include, in particular, issues of selection bias, exposure assessment, and the potential for conflict of interest. Many studies had non-response issues or loss to follow-up. This is mitigated somewhat in that some studies demonstrate that the non-responders did not differ substantially from the included subjects. However, there are may be underlying, unknown differences leading to the non-response, which may in turn lead to bias in effect estimates if these differences are related to both PFAS exposure and the health outcome of interest. For exposure assessment, the largest concern is the issue of timing of exposure. Many of the findings are obtained from cross-sectional studies with associations reported between serum PFAS concentration and measured health outcome, which have the potential to be affected by bias due to reverse causality, unlike a longitudinal study with repeated measurements over time. Some of the studies under review are funded by non-governmental sources, leading to concern over conflict of interest. As described by the Institute of Medicine Roundtable on Environmental Health Sciences, Research, and Medicine Institute of Medicine Roundtable on Environmental Health Sciences and Medicine [112], conflicts of interest do not inherently represent an inadequacy of a publication or signal any level of misconduct, but rather describe a set of circumstances under which researchers may rely on the judgment of an outside force, or be influenced by considerations for such parties. The failure to avoid being compromised by these dependencies and influences may lead to a number of biases, including publication bias or selective reporting of outcomes.

More generally for studies of PFAS, their concentrations are likely to be affected by when, and in whom, they are measured, introducing the potential for exposure misclassification. Measures during pregnancy are taken in maternal serum or cord blood. However, not all PFAS are equally transferred across the placenta [113]. Therefore, concentrations of PFAS in maternal serum are likely to differentially represent fetal PFAS, and there will be differential representation based on specific PFAS and placental transfer ability [113]. As well, maternal blood volume expands during pregnancy, leading to the potential for different periods of pregnancy to have different maternal serum concentrations of PFAS due to blood volume changes. Blood samples may also be taken from either fasting or non-fasting participants, which may make between study comparison of cardiovascular and lipid-related makers difficult. These potential sources of exposure misclassification may attenuate health effect estimates, as they are likely non-differential by outcome.

In addition, the potential exists for non-monotonic dose response curves for PFAS, some of which are known endocrine disrupting compounds. It is possible that lower concentrations/exposures may have a more disruptive effect than high concentrations/exposures, in particular with outcomes connected to the endocrine system such as thyroid function or pubertal development.

Relatedly, the toxicological effects of PFASs as a mixture of potentially dozens to hundreds of compounds are generally unknown [114]. Only a handful of studies covered in this review examined PFASs using a mixture method, and this was either a summed value or a factor representing PFAS exposure [44,52,75,83]. While these studies provide some context for effects of PFASs as a mixture, they are limited in number and not performed for all health outcomes. A summed or representative metric of PFAS exposure also cannot inform researchers about potential interactions between individual PFASs. A more through exploration of how PFASs interact with one another, both in a toxicological manner and in effect measure modification, would help the understanding of how these exposures may or may not lead to adverse health outcomes.

With cross-sectional studies, temporality cannot be established. Therefore, it is not possible to determine whether observed health effects are due to PFAS exposure, or if underlying health conditions lead to a buildup of PFAS. Optimal study design to address this challenge would be longitudinal studies with repeated measurements before and after disease onset, which would establish temporality between PFAS exposure and health effects.

Design of studies involving thyroid disease and thyroid hormone concentrations are complicated by disease status and medication use. Those with known thyroid disease should be separated from those who are disease-free for comparison. Patients with thyroid disease are likely monitored and medicated to have thyroid hormone levels in a therapeutic range and comparisons of T4 or TSH with this population’s PFAS concentration would not be informative. Autoimmune status and iodine sufficiency are also informative when included in models.

Within the published literature, there is an incomplete assessment of pubertal onset in girls. Epidemiologic publications for pubertal onset in girls across PFAS concentrations look at age at menarche, but lack information on thelarche or the onset of female breast development. Breast development has been shown to be sensitive to PFAS exposure in laboratory animals and the dearth of information on this endpoint in developing human populations is an area that could be expanded to allow for better cross-species comparison. Existing cohorts of US girls with data on these pubertal endpoints could be mined to understand these associations. The small number of studies for particular health outcomes limits our ability to draw conclusions based on the body of literature. Even general categories of childhood health outcomes (e.g., cardiometabolic) included only a few studies, though the neurodevelopmental literature has grown substantially even in just the past year. In addition, some of the same outcomes were classified or measured differently in different studies, making direct comparisons across studies more difficult. A focus on particular outcomes of import could help define the literature and direct future research in directions of interest and utility, and prompt more direct mechanistic studies.

## 5. Conclusions

On the basis of our systematic evaluation of PFAS exposure and childhood health outcomes, we observed generally consistent evidence for PFAS’ association with dyslipidemia, immunity including vaccine response and asthma, renal function, and age at menarche. Similar to this evaluation, the National Toxicology Program Office of Health Assessment and Translation recently performed a systematic review on the immunotoxicology associated with exposure to PFOA or PFOS and concluded that PFOA or PFOS is “presumed to be an immune hazard to humans” (NTP, 2016). While the body of literature of PFAS and childhood health outcomes continues to develop, there are several areas that could be addressed. One important consideration is the need for more longitudinal studies where timing of exposure versus outcome can be firmly established. Another important factor to consider in future research is the variability in outcome measurement. Cohesive measurement or definition of health outcomes under study would strengthen conclusions based on the whole of the literature. As exposure to certain PFAS is ubiquitous in the US population, it is important to understand the health effects associated with exposure to the mixtures of PFAS, especially in children.

## Supplementary Materials

The following are available online at www.mdpi.com/1660-4601/14/7/691/s1, Table S1: Search terms used for systematic review, Table S2: Studies of perfluorinated compounds and neurodevelopmental and attention related outcomes, Table S3: Studies of perfluorinated compounds and cardiometabolic related outcomes, Table S4: Studies of perfluorinated compounds and immunity, infection, and asthma related outcomes, Table S5: Studies of perfluorinated compounds and puberty related outcomes, Table S6: Studies of perfluorinated compounds and thyroid related outcomes, Table S7: Studies of perfluorinated compounds and renal related outcomes.
