# Supplementary material for: Exposure to Perfluorinated Alkyl Substances and Health Outcomes in Children: A Systematic Review of the Epidemiologic Literature
Source: Int J Environ Res Public Health. 2017 Jun 27;14(7):691. doi: 10.3390/ijerph14070691 (PMC5551129; doi:10.3390/ijerph14070691)
Supplement: Supplementary file 1 [file ijerph-14-00691-s001.pdf]

International Journal of
Environmental Research and Public Health

MDPI

Supplementary Materials: Exposure to Perfluorinated Alkyl Substances and Health Outcomes in Children: A Systematic Review of the Epidemiologic Literature

Kristen M. Rappazzo, Evan Coffman, and Erin P. Hines

Epidemiologic Evidence of Relationships between Perfluorinated Chemical Exposure and Reproductive or Child Health Outcomes

Table S1. Search terms used for systematic review.

Exposure/PFAS Search Terms	Outcome/Other Search Terms
Perfluorinated	Children
perfluorooctane sulfonate	child
perfluorooctanoate	menarche
polyfluoroalkyl compounds	development
Polyfluoroalkyl chemicals	male reproductive health
Perfluorinated chemicals	testosterone
Perfluorooctanoic acid	reproduction
perfluorooctane sulfonic acid	cord blood
perfluorinated acid	Maternal blood
fluorocarbons	Prenatal exposure
Perfluorinated alkyl substances	Fetal exposure
Perfluorohexane sulfonate	Prenatal exposure delayed effects
perfluoroalkyl acids	mental developmental milestones
fluorinated organic compounds	motor developmental milestones
PFOA	neurodevelopment
PFOS	Attention Deficit Hyperactivity Disorder, ADHD
PFAA	offspring obesity
PFNA	overweight
PFC	serum lipids
PFHxS	Adiposity
PFOSA	Glycemic Control
	immunity
	immune
	eGFR
	kidney function
	T4
	Thyroid disease
	Thyroid hormones
	Thyroid stimulating hormone, TSH

Boolean Search examples

Searches were performed by joining two parenthetical terms with an AND operator. The first term was comprised of PFAS terms linked with OR operators, and the second with health/other terms linked with OR operators. Both “simple” (wherein PFAS terms were limited to PFOA, PFOS, and PFAS or PFC) and full (all PFAS terms) were performed.

Simple search with limited exposure terms, initial search was: (PFOA OR PFOS OR PFAS) AND (child

OR children)

(PFOA[All Fields] OR PFOS[All Fields] OR PFAS[All Fields]) AND ((“child”[MeSH Terms] OR “child”[All Fields]) OR (“child”[MeSH Terms] OR “child”[All Fields] OR “children”[All Fields])) AND (“humans”[MeSH Terms] AND English[lang])

Full search, all terms included

(Perfluorinated[All Fields] OR (“perfluorooctane sulfonic acid”[Supplementary Concept] OR “perfluorooctane sulfonic acid”[All Fields] OR “perfluorooctane sulfonate”[All Fields]) OR (“perfluorooctanoic acid”[Supplementary Concept] OR “perfluorooctanoic acid”[All Fields] OR “perfluorooctanoate”[All Fields]) OR (polyfluoroalkyl[All Fields] AND compounds[All Fields]) OR (Polyfluoroalkyl[All Fields] AND chemicals[All Fields]) OR (Perfluorinated[All Fields] AND chemicals[All Fields]) OR (“perfluorooctanoic acid”[Supplementary Concept] OR “perfluorooctanoic acid”[All Fields]) OR (“perfluorooctane sulfonic acid”[Supplementary Concept] OR “perfluorooctane sulfonic acid”[All Fields]) OR (perfluorinated[All Fields] AND (“acids”[MeSH Terms] OR “acids”[All Fields] OR “acid”[All Fields]) AND (“fluorocarbons”[MeSH Terms] OR “fluorocarbons”[All Fields])) OR (Perfluorinated[All Fields] AND alkyl[All Fields] AND substances[All Fields]) OR ((“perflexane”[Supplementary Concept] OR “perflexane”[All Fields] OR “perfluorohexane”[All Fields]) AND (“alkanesulfonates”[MeSH Terms] OR “alkanesulfonates”[All Fields] OR “sulfonate”[All Fields])) OR (perfluoroalkyl[All Fields] AND (“acids”[MeSH Terms] OR “acids”[All Fields])) OR (fluorinated[All Fields] AND (“organic chemicals”[MeSH Terms] OR (“organic”[All Fields] AND “chemicals”[All Fields]) OR “organic chemicals”[All Fields] OR (“organic”[All Fields] AND “compounds”[All Fields]) OR “organic compounds”[All Fields])) OR PFOA[All Fields] OR PFOS[All Fields] OR PFAA[All Fields] OR PFNA[All Fields] OR PFC[All Fields] OR PFHxS[All Fields] OR (“perfluorooctane sulfonic acid”[Supplementary Concept] OR “perfluorooctane sulfonic acid”[All Fields] OR “pfosa”[All Fields])) AND (eGFR[All Fields] OR ((“kidney”[MeSH Terms] OR “kidney”[All Fields]) AND (“physiology”[Subheading] OR “physiology”[All Fields] OR “function”[All Fields] OR “physiology”[MeSH Terms] OR “function”[All Fields])) OR T4[All Fields] OR (“thyroid diseases”[MeSH Terms] OR (“thyroid”[All Fields] AND “diseases”[All Fields]) OR “thyroid diseases”[All Fields] OR (“thyroid”[All Fields] AND “disease”[All Fields]) OR “thyroid disease”[All Fields]) OR (“thyroid hormones”[MeSH Terms] OR (“thyroid”[All Fields] AND “hormones”[All Fields]) OR “thyroid hormones”[All Fields]) OR (“thyrotropin”[MeSH Terms] OR “thyrotropin”[All Fields] OR (“thyroid”[All Fields] AND “stimulating”[All Fields] AND “hormone”[All Fields]) OR “thyroid stimulating hormone”[All Fields]) OR TSH[All Fields] OR (“attention deficit disorder with hyperactivity”[MeSH Terms] OR (“attention”[All Fields] AND “deficit”[All Fields] AND “disorder”[All Fields] AND “hyperactivity”[All Fields]) OR “attention deficit disorder with hyperactivity”[All Fields] OR (“attention”[All Fields] AND “deficit”[All Fields] AND “hyperactivity”[All Fields] AND “disorder”[All Fields]) OR “attention deficit hyperactivity disorder”[All Fields]) OR (“attention deficit disorder with hyperactivity”[MeSH Terms] OR (“attention”[All Fields] AND “deficit”[All Fields] AND “disorder”[All Fields] AND “hyperactivity”[All Fields]) OR “attention deficit disorder with hyperactivity”[All Fields] OR “adhd”[All Fields]) OR (offspring[All Fields] AND (“obesity”[MeSH Terms] OR “obesity”[All Fields])) OR (“overweight”[MeSH Terms] OR “overweight”[All Fields]) OR ((“serum”[MeSH Terms] OR “serum”[All Fields]) AND (“lipids”[MeSH Terms] OR “lipids”[All Fields])) OR (“adiposity”[MeSH Terms] OR “adiposity”[All Fields]) OR (Glycemic[All Fields] AND (“prevention and control”[Subheading] OR (“prevention”[All Fields] AND “control”[All Fields]) OR “prevention and control”[All Fields] OR “control”[All Fields] OR “control groups”[MeSH Terms] OR (“control”[All Fields] AND “groups”[All Fields]) OR “control groups”[All Fields])) OR (“immunity”[MeSH Terms] OR “immunity”[All Fields]) OR immune[All Fields] OR (“fetal blood”[MeSH Terms] OR (“fetal”[All Fields] AND “blood”[All Fields]) OR “fetal blood”[All Fields] OR (“cord”[All Fields] AND “blood”[All Fields]) OR “cord blood”[All Fields]) OR ((“mothers”[MeSH Terms] OR

"mothers"[All Fields] OR "maternal"[All Fields]) AND ("blood"[Subheading] OR "blood"[All Fields] OR "blood"[MeSH Terms])) OR (("prenatal care"[MeSH Terms] OR ("prenatal"[All Fields] AND "care"[All Fields]) OR "prenatal care"[All Fields] OR "prenatal"[All Fields]) AND exposure[All Fields]) OR (("fetus"[MeSH Terms] OR "fetus"[All Fields] OR "fetal"[All Fields]) AND exposure[All Fields]) OR ("prenatal exposure delayed effects"[MeSH Terms] OR ("prenatal"[All Fields] AND "exposure"[All Fields] AND "delayed"[All Fields] AND "effects"[All Fields]) OR "prenatal exposure delayed effects"[All Fields]) OR (mental[All Fields] AND developmental[All Fields] AND milestones[All Fields]) OR (motor[All Fields] AND developmental[All Fields] AND milestones[All Fields]) OR neurodevelopment[All Fields] OR ("child"[MeSH Terms] OR "child"[All Fields] OR "children"[All Fields]) OR ("child"[MeSH Terms] OR "child"[All Fields]) OR ("menarche"[MeSH Terms] OR "menarche"[All Fields]) OR ("growth and development"[Subheading] OR ("growth"[All Fields] AND "development"[All Fields]) OR "growth and development"[All Fields] OR "development"[All Fields]) OR (("male"[MeSH Terms] OR "male"[All Fields]) AND ("reproductive health"[MeSH Terms] OR ("reproductive"[All Fields] AND "health"[All Fields]) OR "reproductive health"[All Fields])) OR ("testosterone"[MeSH Terms] OR "testosterone"[All Fields]) OR ("reproduction"[MeSH Terms] OR "reproduction"[All Fields])) AND ("humans"[MeSH Terms] AND English[lang])

Guidelines for Making Risk of Bias Determinations in Epidemiologic Studies of Perfluorinated Chemical Exposure and Child Health Outcomes

The following guidelines outline the criteria we used to evaluate the methodological design and implementation of the studies included in our review. The risk of bias criteria in our guidelines were directly adapted from a systematic review of PFOA effects on fetal growth [Johnson et al., 2014], which developed its risk of bias framework from the Cochrane Collaboration's Risk of bias tool and the Agency for Healthcare Research and Quality's criteria [Higgins and Green, 2011; Viswanathan et al., 2012]. The guidelines outline seven potential biases with specific criteria for a low risk of bias determination within each category. Subsequently, there is a general summary of the criteria for higher risks of bias.

1. Selection Bias

   Criteria for a "low risk of bias" judgment:
   - Study participants were recruited/selected from the same population at the same time frame; or:
   - Study participants were not all recruited from the same population, but proportions of participants from each population in each study group are uniform;
     AND:
   - Loss to follow-up in longitudinal studies is minimized and any loss to follow-up is not expected to differ greatly between levels of exposure and/or outcome; and:
   - Other sources of potential selection bias are minimized (e.g., Berkson's bias, nonresponse bias, incidence-prevalence bias, volunteer/self-selection bias)

2. Exposure Assessment

   Criteria for a "low risk of bias" judgment:
   "The reviewers judge that there is low risk of exposure misclassification and any one of the following:
   - There is high confidence in the accuracy of the exposure assessment methods; or:
   - Less-established or less direct exposure measurements are validated against well-established or direct methods"

3. Outcome Assessment

Criteria for a "low risk of bias" judgment:
The reviewers judge that there is low risk of outcome misclassification and any one of the following:
- There is high confidence in the accuracy of the outcome assessment methods (e.g., clinical diagnosis, laboratory testing, sensitive instrument); or:
- Less-established or less direct outcome measurements are validated against well-established or direct methods; or:
- If self- or parental-reported outcomes are being used, there is high confidence in the accuracy of those reports; or:
- If a proxy measure is being used, the outcome being measured provides a reasonable surrogate for the outcome of interest

4. Confounding

Criteria for a "low risk of bias" judgment:
"The study accounted for (i.e., matched, stratified, multivariate analysis or otherwise statistically controlled for) important potential confounders, or reported that potential confounders were evaluated and omitted because inclusion did not substantially affect the results." The determination of specific confounders that should be adjusted for will depend on the details of the individual studies, and evaluation will be subject to the judgment of the reviewer. However, the following is a general list of potentially important confounders:
- Age, Sex, Race, Maternal Age at Delivery, Maternal Education, Family Income, ETS, Maternal Alcohol Use DP, Maternal Smoking DP, and Breast Feeding

5. Missing Data

Criteria for a "low risk of bias" judgment:
"Participants were followed long enough to obtain outcome measurements and any one of the following:
- No missing outcome data; or:
- Reasons for missing outcome data unlikely to be related to true outcome (for survival data, censoring unlikely to be introducing bias); or:
- Missing outcome data balanced in numbers across exposure groups, with similar reasons for missing data across groups; or:
- For dichotomous outcome data, the proportion of missing outcomes compared with observed event risk not enough to have a biologically relevant impact on the intervention effect estimate; or:
- For continuous outcome data, plausible effect size (difference in means or standardized difference in means) among missing outcomes not enough to have a biologically relevant impact on observed effect size; or:
- Missing data have been imputed using appropriate methods"

6. Conflict of Interest

Criteria for a "low risk of bias" judgment:
"The study did not receive support from a company, study author, or other entity having a financial interest in the outcome of the study. Examples include the following:
- Funding source is limited to government, non-profit organizations, or academic grants funded by government, foundations and/or non-profit organizations;

- Chemicals or other treatment used in study were purchased from a supplier;
- Company affiliated staff are not mentioned in the acknowledgements section;
- Authors were not employees of a company with a financial interest in the outcome of the study;
- Company with a financial interest in the outcome of the study was not involved in the design, conduct, analysis, or reporting of the study and authors had complete access to the data;
- Study authors make a claim denying conflicts of interest;
- Study authors are unaffiliated with companies with financial interest, and there is no reason to believe a conflict of interest exists; OR
- All study authors are affiliated with a government agency (are prohibited from involvement in projects for which there is a conflict of interest or an appearance of conflict of interest)."

General Criteria for Subsequent Risk of Bias Judgments

a) ***Probably low risk of bias:***
   - A small portion of the "low risk of bias" criteria is violated, and/or there is relevant information or analyses suggesting that the violated criteria are unlikely to introduce bias.

b) ***Moderate or unclear risk of bias:***
   - A larger portion of the "low risk of bias" criteria has been violated, but there is evidence that the violated criteria are unlikely to introduce bias; or:
   - There is not enough information in the study to determine the risk of bias for a specific category.

c) ***Probably high risk of bias:***
   - The "low risk of bias" criteria are violated, but there is some indication that the violated criteria may not introduce substantial bias.

d) ***High risk of bias:***
   - The "low risk of bias" criteria are violated and there is no evidence that the violated criteria are unlikely to introduce substantial bias.

Table S2. Studies of perfluorinated compounds and neurodevelopmental and attention related outcomes

Study	Study Characteristics	Exposure Matrix	Exposure Contrast ng/mL (Mean or Median)	Summary of Results	Selected Effect Estimates
Hoffman et al. (2010)	Cross-sectional<br>United States<br>(n=571)<br>1999-2000, 2003-2004<br>NHANES	Serum	1 µg/L<br>(PFOA: 4.4)	Positive association with ADHD and PFOA.	PFOA<br>ADHD (OR)<br>1.12 (1.01, 1.23)<br>Medication use adjusted<br>1.19 (0.95, 1.49)
			(PFOS: 22.6)	Increases in PFOS associated with increased odds of ADHD.	PFOS<br>ADHD (OR)<br>1.03 (1.01, 1.05)<br>Adjusted for medication use<br>1.05 (1.02, 1.08)
Stein and Savitz (2011)	Cross-sectional<br>United States<br>(n=10546)<br>2005-2006<br>C8	Serum	PFOA<br>Q1: <13.0<br>Q2: 13.0 -<28.2<br>Q3: 28.2 -<65.3<br>Q4: >65.3	Null or potentially inverse associations with parent-reported ADHD or learning problems and PFOA.	PFOA<br>ADHD (OR)<br>Age 12-15<br>Q1: ref<br>Q2: 1.18 (0.91, 1.53)<br>Q3: 0.93 (0.71, 1.21)<br>Q4: 0.79 (0.60, 1.04)
			PFOS<br>Q1: < 14.8<br>Q2: 14.8-20.1<br>Q3:20.2-27.9<br>Q4: > 27.9	ADHD associated with PFOS with adjustment for medication use. Inverse ORs for learning problems with increasing PFOS.	ADHD (OR), age 12-15<br>Q1: ref<br>Q2: 0.91 (0.70, 1.19)<br>Q3: 0.92 (0.71, 1.21)<br>Q4: 0.99 (0.76, 1.30)<br>ADHD + medication use (OR), age 12-15<br>Q1: ref<br>Q2: 1.40 (0.94, 2.08)<br>Q3: 1.38 (0.92, 2.06)<br>Q4: 1.32 (0.88, 1.99)
			PFHxS<br>Q1: <2.9<br>Q2: 2.9 - 5.1<br>Q3: 5.2 - 10.1<br>Q4: >10.1<br><br>PFNA<br>Q1: <1.2<br>Q2: 1.2 - 1.4	ADHD positively associated with PFHxS. PFNA not associated with ADHD.	PFHxS<br>ADHD (OR), age 12-15<br>Q1: ref<br>Q2: 1.46 (1.10, 1.93)<br>Q3: 1.45 (1.10, 1.91)<br>Q4: 1.53 (1.15, 2.04)<br>ADHD + medication use (OR), age 12-15<br>Q1: ref

			Q3: 1.5 – 2.0<br>Q4: >2.0		Q2: 1.32 (0.87, 1.99)<br>Q3: 1.32 (0.88, 1.97)<br>Q4: 1.42 (0.94, 2.13)
Ode et al. (2014)	Case-control<br>Sweden<br>(n = 206 cases, 206 controls)<br>1978 - 2005	Cord serum	PFOA: 1 (1.80, 1.83)	No associations between ADHD diagnosis and PFOA.	ADHD (OR)<br>PFOA:<br>0.98 (0.92, 1.04)
			PFOS: 1 (6.92, 6.77)	No associations between ADHD diagnosis and PFOS.	PFOS:<br>0.98 (0.94, 1.02)
			PFNA:<br>LOD (0.2)	No associations between ADHD diagnosis and PFNA.	PFNA:<br><LOD: ref<br>>= LOD: 1.1 (0.75, 1.7)
Donauer et al. (2015)	Cohort<br>United States<br>(n = 327)<br>Mar 2003 – Jan 2006<br>Health Outcomes and Measures of the Environment Study	Maternal serum	1-log unit<br>(PFOA: 5.49)	No associations between PFOA and neurobehavioral measures. Positive association with hypotonic status and PFOA, CIs not reported.	PFOA<br>Attention (Beta (SE))<br>0.01 (0.06)<br>Hypotonicity (Beta (SE))<br>0.14 (0.07)<br>Hypotonic status (OR)<br>3.785
			(PFOS: 13.25)	No associations between PFOS and neurobehavioral measures.	PFOS<br>Attention (Beta (SE))<br>0.01 (0.06)<br>Hypotonicity (Beta (SE))<br>0.04 (0.07)<br>Hypotonic status (OR)<br>1.780
Stein et al. (2014)	Cohort<br>United States<br>(n=321)<br>2005-2006, 2009-2010<br>C8	Serum	1 Ln-unit<br>(35.1)	Observed effects for ADHD scores depended upon who was reporting. If mothers, boys had improved scores and girls had worse with increasing PFOA. If teachers, sex differences are less pronounced, and potentially improved scores with increasing PFOA are observed.	PFOA<br>BRIEF score (Δ)<br>Mother survey<br>-0.08 (-1.05, 0.89)<br>Boys only<br>-1.76 (-3.23, -0.29)<br>Girls only<br>1.09 (-0.14, 2.32)<br>Teacher survey<br>-1.32 (-3.10, 0.47)<br>Boys only<br>-2.82 (-4.96, -0.68)<br>Girls only<br>-0.48 (-2.81, 1.85)

Strom et al. (2014)	Cohort<br>Denmark<br>(n=876)<br>1988-2010<br>Danish Fetal Origins 1988	Maternal serum	PFOA<br>T1: <=3.13<br>T2: >3.13–4.39<br>T3: >4.39	Potential inverse associations between PFOA and ADHD, with wide CI. ORs for depression are positive only for the middle tertile and return to null at the highest exposure. No association scholastic achievement score.	PFOA<br>ADHD (OR)<br>T1: ref<br>T2: 0.48 (0.18, 1.28)<br>T3: 0.74 (0.29, 1.87)<br><br>Depression (OR)<br>T1: ref<br>T2: 1.37 (0.85, 2.21)<br>T3: 1.03 (0.61, 1.73)
				Potential inverse association between PFOS and ADHD at highest tertile of exposure. ORs for depression are positive only for the middle tertile and return to null at the highest exposure. No association scholastic achievement score.	PFOS<br>ADHD (OR)<br>T1: ref<br>T2: 1.05 (0.43, 2.53)<br>T3: 0.54 (0.19, 1.53)<br><br>Depression (OR)<br>T1: ref<br>T2: 1.61 (0.99, 2.61)<br>T3: 1.16 (0.69, 1.95)
Hoyer et al. (2015a)	Cohort<br>Greenland, Ukraine, and Poland<br>(n=1106)<br>INUENDO<br>2002-2004	Maternal serum	1 log-unit<br>(PFOA: 1.4)	Log-increases in PFOA associated with behavioral problems and hyperactivity.	PFOA<br>Behavioral problems (OR)<br>1.5 (0.9, 2.6)<br>Hyperactivity (OR)<br>1.6 (0.9, 2.8)
			(PFOS: 10.0)	Log-increases in PFOS associated with increased odds of hyperactivity but not behavioral problems.	PFOS<br>Behavioral problems (OR)<br>1.1 (0.6, 2.0)<br>Hyperactivity (OR)<br>1.7 (0.9, 3.2)
Liew et al. (2015)	Case-control<br>Denmark<br>(cases = 220 for each outcome, controls = 550)<br>1996-2002<br>Danish National Birth Cohort	Maternal plasma	1 ln-unit<br>(PFOA: 4.00)	Potential positive association between in PFOA and ADHD, for both ln-linear and quartile increases. No association with autism.	PFOA<br>ADHD (OR)<br>1.21 (0.84, 1.74)<br>Autism (OR)<br>1.15 (0.68, 1.93)
			(PFOS: 27.40)	Either no or inverse association between PFOS and ADHD, slightly elevated ORs for autism but confidence intervals are very wide.	PFOS<br>ADHD (OR)<br>1.04 (0.70, 1.56)

					Autism (OR)<br>1.21 (0.69, 2.13)
				Other PFASs show primarily inverse or null associations.<br>PFHxS has increasing OR for autism with both ln-linear and quartile increases.<br>PFNA shows positive association with ADHD only with quartile increases.	PFHxS<br>ADHD (OR)<br>1.05 (0.91, 1.20)<br>Autism (OR)<br>1.26 (1.00, 1.58)<br><br>PFNA<br>ADHD (OR)<br>0.99 (0.58, 1.7)<br>Autism(OR)<br>0.84 (0.48, 1.49)
Fei et al. (2008)	Cohort<br>Denmark<br>(n = 1400)<br>1996-2002<br>Danish National Birth Cohort	Plasma from maternal blood during 1st trimester	PFOA<br>Q1: <3.91<br>Q2: 3.91-5.20<br>Q3: 5.21 – 6.96<br>Q4: >6.96	No associations between "developmental milestones" and PFOA. Slight negative association with "sitting without support".	PFOA<br>Walk without support (Hazard ratio (HR))<br>Q1: ref<br>Q2: 1.10 (0.94, 1.28)<br>Q3: 1.04 (0.88, 1.22)<br>Q4: 0.94 (0.80, 1.12)
			PFOS<br>Q1: <26.1<br>Q2: 26.1-33.3<br>Q3: 33.4 – 43.2<br>Q4: >43.2	Generally null associations or negative associations with large confidence intervals between developmental milestones and PFOS. "Sitting without support" had inverse HRs with PFOS.	PFOS<br>Attention (HR)<br>Q1: ref<br>Q2: 1.13 (0.75, 1.69)<br>Q3: 1.05 (0.69, 1.59)<br>Q4: 0.94 (0.61, 1.44)<br>Sitting w/out support (HR)<br>Q1: ref<br>Q2: 0.93 (0.79–1.08)<br>Q3: 0.85 (0.72–0.99)<br>Q4: 0.86 (0.73–1.01)
Chen et al. (2013)	Cohort<br>Taiwan<br>(n=239)<br>2004<br>Taiwan Birth Panel Study	Cord blood	10<br>(PFOA: 2.6)	No association between neurodevelopmental markers and PFOA.	PFOA<br>Comprehensive Developmental Inventory for Infants and Toddlers<br>Overall (Δ score)<br>-2.4 (-8.9, 4.1)<br>Poor-performance, overall (OR)<br>0.6 (0.08, 4.8)
			(PFOS: 7.4)	Odds of poor-performance on developmental test measures are increased with PFOS, though the associations for changes in scores are less pronounced.	PFOS<br>Whole test (Δ score)<br>-2.3 (-5.1, 0.4)<br>Whole test (OR poor performance)

					1.3 (0.7, 2.4)<br>Gross-motor skills (Δ score)<br>-4.9 (-8, -1.7)<br>Gross-motor skills (OR poor performance)<br>2.2 (1.2, 3.9)
Fei and Olsen (2011)	Cohort<br>Denmark<br>(n=1400)<br>1996-2002, 2005-2010<br>Danish National Birth Cohort	Plasma from maternal blood during 1st trimester	PFOA<br>Q1: <3.95<br>Q2: 3.96 – 5.32<br>Q3: 5.33 – 7.11<br>Q4: >7.11	Null associations for PFOA with large confidence intervals, there are some inverse associations.	PFOA<br>Strengths and Difficulties Questionnaire/Developmental Coordination Disorder Questionnaire<br>Total Difficulties (OR)<br>Q1: ref<br>Q2: 0.56 (0.27, 1.19)<br>Q3: 0.36 (0.15, 0.82)<br>Q4: 0.91 (0.43, 1.92)
			PFOS<br>Q1: <26.5<br>Q2: 26.5-34.3<br>Q3: 34.4-44.3<br>Q4: >44.3	No association with PFOS and behavioral and motor coordination problems.	Total difficulties (OR)<br>Q1: ref<br>Q2: 0.95 (0.47, 1.91)<br>Q3: 0.56 (0.25, 1.22)<br>Q4: 0.92 (0.45, 1.87)
Lien et al. (2016)	Cohort<br>Taiwan<br>(n=1526)<br>2004-2005<br>Taiwan Birth Panel Study and the Taiwan Early-Life Cohort	Cord blood	PFOA<br>50th percentile: 0.75<br>75th: 2.09<br>90th: 3.78	No associations between PFOA and neurobehavioral symptoms related to ADHD measured through the Swanson, Nolan, and Pelham IV scale (SNAP-IV), the Child Behavior Checklist (CBCL), and the Strengths and Difficulties Questionnaire (SDQ) questionnaires.	SNAP-IV<br>Inattention (Δ score)<br><br>PFOA<br><50th percentile: ref<br>50th-74th: -2.29 (-3.09, -1.48)<br>75th-89th: -1.8 (-3.04, -0.55)<br>>90th: -1.39 (-2.84, 0.07)
			PFOS<br>50th percentile: 3.7<br>75th: 5.76<br>90th: 8.45	No associations between PFOS concentrations and neurobehavioral symptoms.	PFOS<br><50th percentile: ref<br>50th-74th: 0.4 (-0.81, 1.6)<br>75th-89th: -0.51 (-1.95, 0.92)<br>>90th: -0.69 (-2.43, 1.04)
			PFNA<br>50th percentile: 1.29<br>75th: 4.69<br>90th: 14.2<br><br>PFUA<br>50th percentile: 2.86<br>75th: 11.7	Increasing percentile categories of PFNA were associated with Inattention, hyperactivity/impulsivity, and oppositional defiant disorder measured by SNAP-IV. No associations between PFNA and neurobehavioral symptoms measured by CBCL or SDQ. No associations between PFUA and neurobehavioral symptoms.	PFNA<br><50th percentile: ref<br>50th-74th: -0.14 (-1.33, 1.06)<br>75th-89th: -1.03 (-2.33, 0.26)<br>>90th: -2.11 (-3.99, -0.23)<br><br>PFUA<br><50th percentile: ref

			90th: 21.18		50th-74th: -0.52 (-1.56, 0.52)<br>75th-89th: -1.38 (-2.75, -0.01)<br>>90th: -0.84 (-2.34, 0.66)
Quaak et al. (2016)*	Cohort<br>The Netherlands<br>(n=76)<br>2011-2013<br>Linking Maternal Nutrition to Child Health cohort	Cord blood	PFOA<br>1st tertile: <0.64<br>2nd: 0.64 – 1.00<br>3rd: >1.00	Potential associations between externalizing behavior, measured by the Child Behavior Checklist (CBCL) and higher tertiles of PFOA. No associations with ADHD scale.	Externalizing behavior scale (Δ score)<br>PFOA<br>1st tertile: ref<br>2nd: -3.33 (-7.65, 0.29)<br>3rd: -2.3 (-6.88, 1.55)
			PFOS<br>1st tertile: <1.2<br>2nd: 1.2 – 1.8<br>3rd: >1.8	No associations between externalizing behavior or ADHD scales and PFOS	PFOS<br>1st tertile: ref<br>2nd: -1.23 (-5.68, 3.85)<br>3rd: -2.43 (-6.55, 1.93)
			Sum PFAS<br>1st tertile: <0.64<br>2nd: 0.64 – 1.00<br>3rd: >1.00	Potential associations between increasing tertiles of summed PFAS and ADHD scale and CBCL.	ADHD scale (Δ)<br>1st tertile: ref<br>2nd: -0.72 (-1.96, 0.59)<br>3rd: -0.99 (-2.14, 0.18)<br><br>Externalizing behavior scale (Δ score)<br>1st tertile: ref<br>2nd: -1.59 (-5.27, 2.5)<br>3rd: -3.31 (-6.55, 0.28)
Gump et al. (2011)	Cross-sectional<br>United States<br>(n=83)<br>NR	Blood	1 standard deviation<br><br>PFOA: 1.30	Potential associations with lower response inhibition and increasing PFOA.	PFOA<br>Inter-response times (Δ)<br>0-5 min bin<br>-0.03 [-0.24, 0.18]<br>6-10 min bin<br>-0.11 [-0.34, 0.11]<br>11-15 min bin<br>-0.20 [-0.41, 0.01]<br>16-20 min bin<br>-0.17 [-0.39, 0.05]
			PFOS: 6.09	PFOS associated with lower response inhibition.	PFOS<br>Inter-response times (Δ)<br>0-5 min bin<br>-0.05 [-0.26, 0.16]<br>6-10 min bin<br>-0.18 [-0.40, 0.04]<br>11-15 min bin<br>-0.25 [-0.46, -0.04]<br>16-20 min bin

					-0.20 [-0.42, 0.02]
			PFNA: 0.56<br>PFDA: 0.13<br>PFHxS: 8.03<br>PFOSA: 0.57	PFASs generally associated with lower response inhibition.	PFNA<br>Inter-response times (Δ)<br>0-5 min bin<br>-0.07 (-0.29, 0.14)<br>6-10 min bin<br>-0.24 (-0.46, -0.02)<br>11-15 min bin<br>-0.15 ([-0.38, 0.07)<br>16-20 min bin<br>-0.05 (-0.28, 0.18)
Stein et al. (2013)	Cohort<br>United States<br>(n=320)<br>2005-2006, 2009-2010<br>C8	Serum	1 Ln-unit<br>(15.3)	No associations with PFOA and neuropsychological tests	PFOA<br>IQ (Δ)<br>0.83 (-0.13, 1.79)<br>Word Reading Score (Δ)<br>0.5 (-0.4, 1.41)
Forns et al. (2015)	Cohort<br>Norway<br>(n=843-896)<br>2003-2009<br>HUMIS	Breast milk, 1 month post delivery	PFOA<br>IQR:37<br>Median: 40	No associations for abnormal score (AS) in cognitive and psychomotor development with higher PFOA. No associations between behavioral problems and PFOA.	PFOA<br>AS, 6 months (OR)<br>Per IQR: 1.05 (0.77, 1.44)<br>>Median: 1.14 (0.71, 1.80)<br><br>AS, 24 months (OR)<br>Per IQR: 1.00 (0.78, 1.28)<br>>Median: 1.25 (0.81, 1.95)
			PFOS<br>IQR:77<br>Median: 110	No associations for abnormal score in cognitive and psychomotor development with higher PFOS. No associations between behavioral problems and PFOS.	PFOS<br>AS, 6 months (OR)<br>Per IQR: 0.96 (076, 1.20)<br>>Median: 0.93 (0.60, 1.44)<br><br>AS, 24 months (OR)<br>Per IQR: 0.93 (0.74, 1.17)<br>>Median: 0.99 (0.64, 1.52)
Wang et al. (2015)	Cohort<br>Taiwan<br>(n = 120)<br>Dec 2000 – Nov 2001	Maternal serum	1 log-2 unit<br>(PFOA: 2.50)	No association between PFOA and IQ.	Full Scale IQ (Δ score)<br>PFOA<br>1.2 (-1.0, 3.5)
	Taiwan Maternal and Infant Cohort Study		(PFOS: 13.25)	No association between PFOS and IQ.	PFOS<br>-1.9 (-4.3, 0.5)
			(Ranges from 0.38 to 3.42)	No associations between other PFASs and IQ.	PFNA<br>-0.2 (-2.1, 1.7)

					PFHxS<br>0.4 (-2.4, 3.1)
Goudarzi et al. (2016)	Cohort<br>Japan<br>(n = 422)<br>July 2002 - Oct 2005<br>Hokkaido Study on Environment and Children's Health	Maternal serum	1 log-10-unit<br>(PFOA: 1.2)	No association between PFOA and mental development index (MDI) or psychomotor developmental index (PDI) at 6 or 18 months. Small negative association with MDI in girls at 6 months, but does not persist.	MDI at 6 months (Δ score)<br><br>PFOA<br>Total: −0.045 (−4.33 to 2.56)<br>Boys: 0.110 (−3.31 to 7.14)<br>Girls: −0.296 (−11.96 to −0.682)
			(PFOS: 5.7)	No Association between PFOS and MDI or PDI at 6 or 18 months.	PFOS<br>Total: 0.018 (−4.52 to 5.59)<br>Boys: −0.141 (−11.26 to 3.45)<br>Girls: 0.072 (−5.19 to 9.38)
Vuong et al. (2016)	Cohort<br>United States<br>(n = 256)<br>Mar 2003 – Feb 2006<br>Health Outcomes and Measures of the Environment Study	Maternal serum	1 ln-unit<br>(PFOA: 5.4)	No association between PFOA and behavioral regulation, metacognition, or global executive function.	Poorer global executive function (OR)<br><br>PFOA<br>1.06 (-1.33, 3.45)
			(PFOS: 13.2)	PFOS associated with increased odds of poorer behavioral regulation, metacognition, and global executive function.	PFOS<br>3.38 (0.86, 5.9)
			(Ranges from 0.1 to 3.6)	No associations between PFHxS, PFNA, or PFDeA and neurodevelopmental outcomes.	PFHxS<br>1.36 (-0.41, 3.12)

*: Study that examined PFAS as a mixture

Table S3. Studies of perfluorinated compounds and cardiometabolic related outcomes.

Study	Study Characteristics	Exposure matrix	Conc ng/mL	Summary of results	Selected effect estimates
Andersen et al. (2010)	Cohort<br>Denmark<br>(n=1400)<br>1996-2002<br>Danish National birth cohort	Maternal blood	1	No meaningful associations between PFOA and weight, height or BMI at 5 or 12 months of age.	PFOA<br>Weight at 5 months (Δg)<br>-9.4 (-28.6, 9.9)<br>Height at 12 months (Δcm)<br>0.049 (-0.026, 0.124)
				No association between PFOS and weight, height or BMI at 5 or 12 months of age.	PFOS<br>Weight at 5 months (Δg)<br>-0.8 (-4.2, 2.6)<br>Height at 12 months (Δcm)<br>0.010 (-0.003, 0.023)
Andersen et al. (2013)	Cohort<br>Denmark<br>(n=1400)<br>1996-2002<br>Danish National birth cohort	Plasma	1<br>(PFOA: 5.12, 5.31)	No associations between prenatal PFOA and BMI, waist circumference, or overweight status at age 7.	BMI at age 7 (Δ) PFOA<br>Boys: -0.049 (-0.117, 0.02)<br>Girls: -0.019 (-0.098, 0.058)
			PFOS<br>Q1: <25.5<br>Q2: 25.6 – 33.5<br>Q3: 33.6 – 43.4<br>Q4: >43.4	ORs of overweight potentially inverse in both boys and girls with increasing PFOS. BMI and waist circumference at age 7 not associated with PFOS.	BMI at 7 (Δ) PFOS<br>Boys: -0.008 (-0.016, 0.009)<br>Girls: -0.005 (-0.019, 0.008)<br>Overweight (OR),<br>PFOS, girls<br>Q1: ref<br>Q2: 1.04 (0.45, 2.41)<br>Q3: 0.87 (0.37, 2.02)<br>Q4: 0.60 (0.24, 1.49)
Maisonet et al. (2012)	Cohort<br>England<br>(n=447)<br>1991-1992<br>ALSPAC	Maternal serum	T1: <3.1<br>T2: 3.1-4.4<br>T3: >4.4	No association between weight at 20 months and maternal PFOA.	PFOA<br>Weight at 20 months (Δg)<br>T1: ref<br>T2: -174.31 (-550.55, 201.94)<br>T3: 9.48 (-405.48, 424.44)<br>Adjusted for birth weight & height at 20 months<br>T1: ref<br>T2: -184.21 (-465.90, 97.48)<br>T3: 128.40 (-180.94, 437.74)
			T1: <16.6<br>T2: 16.6-23.0<br>T3: >23.0	Increase in weight at 20 months associated with increasing maternal PFOS.	PFOS<br>Weight at 20 months (Δg)<br>T1: ref<br>T2: 100.93 (-282.81, 484.68)<br>T3: 364.35 (-15.14, 743.83)

					Adjusted for birth weight & height at 20 months<br>T1: ref<br>T2: 310.64 (27.19, 594.08)<br>T3: 579.82 (301.40, 858.25)
			PFHxS<br>T1: <1.3<br>T2: 1.3-2.0<br>T3: >2.0	No association between weight at 20 months associated with increasing maternal PFHxS.	PFHxS<br>Weight at 20 months (Δg)<br>T1: ref<br>T2: -31.84 (-416.87, 353.20)<br>T3: 62.86 (-326.68, 452.40)<br>Adjusted for birth weight & height at 20 months<br>T1: ref<br>T2: 12.33 (-275.56, 300.22)<br>T3: 115.4 (-176.69, 407.50)
Wang et al. (2016)	Cohort<br>Taiwan<br>(n = 223)<br>2000 – 2012<br>Taiwan Maternal and Infant Cohort Study	Maternal serum, 3rd trimester	1 ln-unit<br>(PFOA: 1.98)	No association between PFOA and weight or height z-score.	Height z-score (Δ score) PFOA<br>Girls: -0.15 (-0.38, 0.08)<br>Boys: 0.01 (-0.24, 0.25)
			(Ranges from 0.28 to 2.89)	PFDeA, PFUnDA, and PFDoDA associated with decreased height z-score in girls. PFNA potentially negatively associated with height in both boys and girls. No associations with weight in boys or girls.	PFNA<br>Girls: -0.21 (-0.42, 0)<br>Boys: -0.15 (-0.37, 0.08)<br><br>PFDeA<br>Girls: -0.52 (-0.8, -0.24)<br>Boys: -0.05 (-0.34, 0.23)
Braun et al. (2016)	Cohort<br>United States<br>(n = 256)<br>Mar 2003 – Feb 2006<br>Health Outcomes and Measures of the Environment Study	Maternal serum	PFOA<br>1st tertile:<br>0.5-4.2ng/mL<br>2nd tertile:<br>4.3-6.4ng/mL<br>3rd tertile:<br>6.6-25 ng/mL	Non-linear associations between PFOA and BMI z-score, waist circumference and body fat percentage at 8 years, with middle tertile showing higher odds. Positive ORs for overweight/obesity and PFOA.	PFOA<br>Body fat percent (Δ)<br>1: ref<br>2: 3.6 (1.8, 5.5)<br>3: 1.5 (-0.4, 3.4)<br><br>Overweight/Obese (RR)<br>1: ref<br>2: 1.84 (0.97, 3.5)<br>3: 1.54 (0.77, 3.07)
			PFOS<br>1: 0.6 (0.1-0.7)<br>2: 0.9 (0.8-1.0)	No association between PFOS and child adiposity at age 8.	BMI z-score (Δ score)<br>PFOS<br>1: ref

			3: 1.3 (1.1-2.9)		2: -0.12 (-0.43, 0.19)<br>3: -0.05 (-0.36, 0.26)
			PFHxS<br>1: 0.7 (0.1-0.9)<br>2: 1.4 (1.0-1.9)<br>3: 2.9 (2.0-33)	No associations between PFNA or PFHxS and adiposity at age 8.	PFHxS<br>1: ref<br>2: 0.22 (-0.10, 0.54)<br>3: 0.12 (-0.21, 0.45)
Halldorsson et al. (2012)	Cohort<br>Denmark<br>(n = 665)<br>1988-89, 2008-2009	Serum	PFOA:<br>Q1: <2.8<br>Q2: 2.8-<3.7<br>Q3: 3.7-4.8<br>Q4: >4.8<br><br>1 Log-unit	Divergent ORs for males (null) and females (positive) for overweight and waist circumference at highest PFOA quartile exposures. Positive associations with %change insulin and leptin, and negative associations with adiponectin and log-unit increases of PFOA.	Overweight (OR) PFOA:<br>Female:<br>Q1: ref<br>Q2: 1.5 (0.6, 3.5)<br>Q3: 2 (0.9, 4.7)<br>Q4: 3.1 (1.4, 6.9)<br>Male:<br>Q1: ref<br>Q2: 1.2 (0.6, 2.6)<br>Q3: 1.0 (0.4, 2.2)<br>Q4: 1.1 (0.5, 2.6)<br>Leptin (% change) PFOA:<br>Female: 4.8 (0.5, 9.4)<br>Male: 4.5 (-2.6, 12.1)
			PFOS:<br>Q1: <12.4<br>Q2: 12.4-21.5<br>Q3: 21.5-30.6<br>Q4: >30.6	PFOS in gestational not associated with anthropometric measures in 20 year olds	NR
Hoyer et al. (2015b)	Cohort<br>Greenland and Ukraine<br>(n = 1,023)<br>May 2002 to Feb 2004<br>INEUNDO	Maternal plasma	1 log-unit<br>(PFOA: 1.0 – 1.8)	Potential positive association between PFOA and overweight status at 5-9 years. Log-increases in PFOA associated with increased odds of waist-to-hip ratio >0.5 at 5-9 years of age.	PFOA<br>Overweight (OR)<br>1.11 (0.88, 1.38)<br>Waist-to-hip ratio >0.5 (OR)<br>1.30 (0.97, 1.74)
			(PFOS: 5.0 – 20.2)	PFOS not associated with overweight status, but positive ORs observed for waist-to-hip ratio >0.5 at 5-9 years.	PFOS<br>Overweight (OR)<br>0.97 (0.78, 1.21)<br>Waist-to-hip ratio >0.5 (OR)<br>1.38 (1.05, 1.82)
Timmermann et al. (2014)	Cross-sectional<br>Denmark<br>(n=590)<br>1997<br>EYHS	Serum	10<br>(PFOA: 9.3)	Positive associations between markers of glycemic control and PFOA observed only in overweight strata, though large confidence intervals. No associations with adiposity.	Insulin (% change) PFOA<br>Normal weight: 0.7 (-3.6, 5.2)<br>Overweight: 16.2 (5.2, 28.3)<br>BMI (% change) PFOA<br>-2.6 (-25.8, 28)

			(PFOS: 41.5)	Increased levels of glycemic control markers associated with PFOS among overweight subjects. No associations between anthropometric measures and PFOS.	Insulin (% change) PFOS<br>Normal weight<br>0.8 (-3.2, 5)<br>Overweight<br>16.2 (5.2, 28.3)
Geiger et al. (2014a)*	Cross-sectional<br>United States<br>(n=815)<br>1999-2008<br>NHANES	Serum	1 Log-unit<br>(PFOA: 4.2)	Increased LDL-C and total cholesterol, and decreased HDL-C associated with log-unit and increases in PFOA.	PFOA<br>Total cholesterol (Δ): 4.55 (0.90, 8.20)<br>LDL-C: 5.75 (2.16, 9.33)<br>HDL-C: -1.52 (-3.02, -0.03)
			(PFOS: 17.7)	Increased LDL-C associated with log-unit increases in PFOS. No association with HDL-C, or triglycerides.	PFOS<br>Total cholesterol (Δ): 0.06 (0.02, 0.10)<br>LDL-C: 4.28 (1.60, 6.95)<br>Triglycerides: -1.85 (-5.61, 1.91)
				Increased LDL-C, HDL-C total cholesterol, and triglycerides associated with log-unit increases in PFAS.	PFAS<br>LDL-C (Δ) 1.48 (1.15, 1.90)<br>HDL-C: 1.03 (0.70, 1.53)<br>Triglycerides 0.90 (0.56, 1.43)
Lin et al. (2011)	Cohort<br>Taiwan<br>(n = 287)<br>2006-2008	Serum	PFOA:<br><50th percentile: 0.75 – 2.37<br>50-74th: 2.39 – 5.92<br>75-89th: 6.01 – 9.62<br>>90th: >9.62	No associations between increasing PFOA and adiponectin, glucose, insulin, HOMA-IR, HDL-C, TG, or CRP concentrations.	NR - Only p for trend reported
				No associations between PFOS and metabolic indicators.	
				Log-increases in serum PFNA associated with log-increases in adiponectin. Other compounds had null associations.<br><br>No associations between PFUA and metabolic indicators.	
Frisbee et al. (2010)	Cross-sectional<br>United States<br>(n = 12,476)<br>2005-2006<br>C8	Serum	Quintile concentrations not reported.<br>Mean (SD)<br>PFOA: 69.2 (111.9)	Associations with abnormal LDL cholesterol and fasting triglycerides (non-linear). No association with HDL cholesterol	Abnormal LDL-C (OR)<br>PFOA<br>Q1: ref<br>Q2: 1.2 (1.0, 1.5)<br>Q3: 1.2 (1.0, 1.4)<br>Q4: 1.2 (1.0, 1.4)<br>Q5: 1.4 (1.2, 1.7)

			PFOS: 22.7 (12.6)	Positive associations for LDL and total cholesterol associated with PFOS. Negative associations for HDL cholesterol, and near null for fasting triglycerides.	HDL-cholesterol (POR)<br>PFOS<br>Q1: ref<br>Q2: 0.9 (0.8, 1.1)<br>Q3: 0.8 (0.7, 1.0)<br>Q4: 0.8 (0.7, 0.9)<br>Q5: 0.7 (0.6, 0.9)<br>LDL-cholesterol (POR)<br>PFOS<br>Q1: ref<br>Q2: 1.2 (1.0, 1.5)<br>Q3: 1.2 (1.0, 1.5)<br>Q4: 1.3 (1.1, 1.6)<br>Q5: 1.6 (1.3, 1.9)
Maisonet et al. (2015b)	Cohort, subset<br>Avon, UK<br>(n = 111, age 7 n = 88, age 15)<br>1991-1992, recruitment<br>ALSPAC	Maternal serum	NR	Reported positive, though non-linear and non-monotonic, associations with total cholesterol and LDL-cholesterol with PFOA and PFOS. Results were reported from unadjusted models.	Reported as figures. Contrasts not reported.
Lin et al. (2009)	Cross-sectional<br>United States<br>(n=474)<br>1999-2000<br>NHANES	Serum	1 Log-unit<br>(log PFOA: 1.51)	No associations between PFOA and glucose homeostasis and indicators of metabolic syndrome. ORs for metabolic syndrome and components were null or negative, with wide confidence intervals.	Glucose (Δ) PFOA<br>-0.03 (-0.13, 0.07)<br>Log-insulin (Δ) PFOA<br>0.08 (-0.57, 0.21)
			(log PFOS: 3.11)	Log HOMA-IR concentrations positively associated with log-increases in PFOS. Potential positive associations for log insulin and log B-cell concentrations with PFOS. No association for glucose concentration and PFOS.<br>ORs for metabolic syndrome and components were null or negative, with wide confidence intervals.	Glucose (Δ) PFOS<br>-0.03 (-0.1476, 0.0876)<br>Log insulin (Δ) PFOS<br>0.15 (-0.0068, 0.3068)<br>Log HOMA-IR (Δ) PFOS<br>0.15 (0.0128, 0.2872)<br>Log B-cell PFOS<br>0.13 (-0.0464, 0.3064)
			(log PFHS: 0.95)<br>(log PFNA: -0.35)	Negative associations between PFNA and log-insulin, log-HOMA, and log-B-cell	Glucose (Δ) PFNA<br>0.07 (-0.01, 0.15)<br>Log insulin (Δ) PFNA

				concentrations; no associations with glucose. Positive association between PFHS and log-insulin; null associations otherwise. Log PFNA concentration increases associated with increased ORs for glucose >= 5.55 mmol/l or a self-report of taking antihyperglycemic medications. Other PFNA and PFHS associations null or negative.	-0.10 (-0.20, 0.00)<br>Log HOMA-IR (Δ) PFNA<br>-0.08 (-0.16, 0.00)<br>Log B-cell, PFNA<br>-0.12 (-0.24, 0.00)
Zeng et al. (2015)	Cross-sectional<br>Taiwan<br>(n=225)<br>2009-2010<br>Genetic and Biomarkers study for Childhood Asthma (GBCA)	Serum	1 Ln-unit<br>(PFOA: 0.92, 1.1)	Ln-unit increases in PFOA were associated with increases in total cholesterol, LDL-C, and triglyceride concentrations.	PFOA<br>Total-C (Δ)<br>6.57 (2.72, 10.42)<br>HDL-C (Δ)<br>-1.56 (-3.2, 0.08)<br>LDL-C (Δ)<br>4.66 (1.67, 7.65)<br>Triglycerides (Δ)<br>19.63 (14.82, 24.34)
			(PFOS: 32.4, 34.2)	Ln-unit increases in PFOS were associated with increases in total cholesterol, LDL-C, and triglyceride concentrations, though increases were small.	PFOS<br>Total-C (Δ)<br>0.31 (0.18, 0.45)<br>HDL-C (Δ)<br>-0.01 (-0.07, 0.05)<br>LDL-C (Δ)<br>0.28 (0.18, 0.38)<br>Triglycerides (Δ)<br>0.19 (0, 0.38)
			(Ranges from 0.4 to 30.7)	Ln-unit increases in PFNA were associated with increases in total cholesterol, LDL-C, and triglyceride concentrations. Lipid levels were not associated with PFBS, PFDA, PFDoA, PFHxA, PFHxS, or PFTA.	PFNA<br>Total-C (Δ)<br>12.92 (0.73, 25.1)<br>HDL-C (Δ)<br>-2.35 (-7.49, 2.79)<br>LDL-C (Δ)<br>9.63 (0.2, 19.06)<br>Triglycerides (Δ)<br>23.01 (6.49, 39.52)<br><br>PFDA<br>Total-C (Δ)<br>-1.29 (-9.01, 6.42)<br>HDL-C (Δ)

					-1.12 (-4.4, 2.05)<br>LDL-C (Δ)<br>-0.56 (-6.53, 5.41)<br>Triglycerides (Δ)<br>0.57 (-9.97, 11.11)
Geiger et al. (2014b)	Cross-sectional<br>United States<br>(n=1655)<br>1999-2000, 2003-2008<br>NHANES	Serum	1 Ln-unit<br>(PFOA: 4.4)	No association between changes in systolic (SBP) or diastolic (DBP) blood pressure and PFOA. Odds for hypertension were decreased with increases in PFOA.	PFOA<br>SBP (ΔmmHg):<br>-1.22 (-2.74, 0.31)<br>DBP (ΔmmHg):<br>0.36 (-0.99, 1.71)<br>Hypertension (OR):<br>0.76 (0.53, 1.10)
			(PFOS: 18.4)	No association between SBP or DBP and PFOS. Odds for hypertension were decreased with increases in PFOS.	PFOS<br>SBP (ΔmmHg):<br>-0.04 (-1.19, 1.12)<br>DBP (ΔmmHg):<br>0.47 (-0.81, 1.74)<br>Hypertension (OR):<br>0.83 (0.58, 1.19)
Lin et al. (2013a)	Cross-sectional<br>Taiwan<br>(n=228)<br>2006-2008<br>Young Taiwanese Cohort Study	Plasma	PFOS<br>Q1: ≤5.41<br>Q2: 5.42 - 8.65<br>Q3: 8.66 -13.52<br>Q4: >13.52	Increases in mean carotid artery intima-media thickness with increasing quartiles of PFOS.	Reported as mean (SD) and p for trend.

*: Study that examined PFAS as a mixture

Table S4. Studies of perfluorinated compounds and immunity, infection, and asthma related outcomes.

Study	Study Characteristics	Exposure Matrix	Conc ng/mL	Summary of Results	Selected Effect Estimates
Asthma/allergy/infection					
Dong et al. (2013)	Case-control Taiwan (n = 231 asthmatic, 225 non-asthmatic children) 2009-2010 Genetic and Biomarkers study for Childhood Asthma (GBCA)	Serum	PFOA Median cases: 1.2 Median control: 0.5	Positive associations and increasing trend observed for asthma and increasing PFOA	Asthma (OR)<br><br>PFOA<br>Q1: ref<br>Q2: 1.58 (0.89, 2.8)<br>Q3: 2.67 (1.49, 4.79)<br>Q4: 4.05 (2.21, 7.42)
			PFOS Median cases: 33.9 Median control: 28.9	Asthma associated with higher concentrations of PFOS.	PFOS<br>Q1: ref<br>Q2: 1.96 (1.11, 3.47)<br>Q3: 1.32 (0.75, 2.32)<br>Q4: 2.63 (1.48, 4.69)
				Asthma positively associated with PFBS, PFDA, PFDoA, PFHxS, and PFNA. Negative association with PFTA.	PFHxS<br>Q1: ref<br>Q2: 1.12 (0.66, 1.91)<br>Q3: 2.63 (1.54, 4.49)<br>Q4: 3.30 (1.92, 5.67)
Humblet et al. (2014)	Cross-sectional United States (n = 1877) 1999-2000, 2003-2008 NHANES	Serum	1 (PFOA: 4.0)	Positive OR between PFOA and ever asthma. No association with current asthma or wheeze.	Ever asthma (OR)<br>PFOA<br>1.06 (1.00, 1.11)
			(PFOS: 16.8)	No association between ever or current asthma, wheeze and PFOS.	PFOS<br>0.99 (0.96, 1.02)
			(PFNA: 0.8 PFHxS: 2.0)	Potential positive association between ever asthma and PFNA. No association between PFNA and ever asthma or wheeze. No associations between PFHS and ever or current asthma and wheeze.	PFNA<br>1.05 (0.89, 1.23)<br>PFHS<br>0.99 (0.98, 1.01)
Smit et al. (2015)*	Cohort Greenland & Ukraine (n = 1024) 2002-2004 INUENDO	Maternal plasma	1 Standard deviation	No associations between standard deviation increases in factors representing PFASs.	Factor representing PFOA, PFHpA, and PFOS<br><br>Ever asthma (OR)<br>0.83 (0.63–1.10)

					Ever eczema (OR)<br>0.94 (0.77–1.14)<br>Ever wheeze (OR)<br>0.91 (0.75–1.11)<br>Current wheeze (OR)<br>1.05 (0.82–1.35)<br>Current eczema (OR)<br>0.94 (0.73–1.22)
Stein et al. (2016)	Cross-sectional<br>United States<br>(n = 640)<br>1999-2000, 2003-2004<br>NHANES	Serum	1 Log(2)-unit<br>(PFOA: 0.97, 1.79)	PFOA increases associated with rhinitis and potentially with asthma and allergy. No association with wheeze.	Asthma (OR)<br><br>PFOA<br>1.28 (0.81, 2.04)
			(PFOS: 4.88, 20.6)	PFOS increases potentially associated with rhinitis and asthma, no association with wheeze or allergy.	PFOS<br>1.20 (0.88, 1.63)
			(PFNA: 0.62, 0.73<br>PFHxS: 1.53, 2.14)	PFNA increases potentially associated with asthma, allergy, and rhinitis.<br>No evidence for associations between PFHxS increases and odds of asthma, wheeze, allergy, or rhinitis.	PFNA<br>1.26 (0.79, 2.01)<br><br>PFHxS<br>0.98 (0.51, 1.87)
Zhu et al. (2016)	Cross-sectional<br>Taiwan<br>(n = 231 asthmatic children, 225 non-asthmatic children)<br>2009-2010<br>Genetic and Biomarkers study for Childhood Asthma (GBCA)	Serum	(PFOA: 1.00)	Increasing quartiles of PFOA were associated with increasing odds of asthma.	Results reported as figures.
			(PFOS: 33.39)	PFOS concentrations were associated with odds of asthma in males, but not in females.	
			(Ranges from 0.48 to 2.10)	PFBS, PFDA, PFHxS, PFNA were all associated with odds of asthma. There is some potential for divergent effects based on sex (e.g., PFBS).	

Qin et al. (2017)	Cross-sectional<br>Taiwan<br>(n=132 asthmatic children, 186 non-asthmatic children)<br>2009-2010<br>Genetics and Biomarkers study for Childhood Asthma	Serum	1 ln-unit<br><br>Medians in case/controls<br>PFOA: 1.02/0.50	Increasing ln-PFOA associated with increasing odds of asthma, forced expiratory volume in 1 s (FEV1) and forced expiratory flow 25–75% (FEF25-75) but not forced vital capacity (FVC) or peak expiratory flow (PEF) in children with asthma. No associations between lung function and PFOA in children without asthma.	PFOA<br>Asthma (OR):<br>2.76 (1.82, 4.17)<br>FEV1 (ΔL):<br>-0.10 (-0.19, -0.02)<br>FVC (ΔL):<br>-0.07 (-0.17, 0.03)
			PFOS: 31.51/28.83	Increasing ln-PFOS associated with increasing odds of asthma, and decreasing FEV1 and FVC in children with asthma. No association between PEF and FEF25-75 in children with asthma, or lung function metrics in children without asthma.	PFOS<br>Asthma (OR):<br>1.30 (1.00, 1.69)<br>FEV1 (ΔL):<br>-0.10 (-0.19, -0.02)<br>FVC (ΔL):<br>-0.06 (-0.10, -0.01)
			PFBS: 0.48<br>PFDA: 1.13/0.93<br>PFHxA: 0.20/0.18<br>PFHxS: 2.38/1.07<br>PFNA: 1.00/0.80<br>PFTA: 2.65/4.52	Increasing PFHxS and PFNA concentrations were associated with decreases in lung function in asthmatic children. No PFASs were associated with lung function in non-asthmatic children.	FEV1 (ΔL):<br>PFBS: 1.06 (0.93, 1.20)<br>PFDA: 1.24 (0.97, 1.58)<br>PFHxA: 0.99 (0.80, 1.21)<br>PFHxS: 2.14 (1.48, 3.11)<br>PFNA: 1.61 (1.12, 2.31)<br>PFTA: 1.14 (1.06, 1.23)<br><br>FEV1 (ΔL)<br>PFBS: 0.10 (-0.11, 0.30)<br>PFDA: -0.05 (-0.15, 0.04)<br>PFHxA: -0.03 (-0.10, 0.04)<br>PFHxS: -0.10 (-0.17, -0.04)<br>PFNA: -0.20 (-0.34, -0.06)<br>PFTA: -0.02 (-0.05, 0.01)
Fei et al. (2010)	Cohort<br>Denmark<br>(n=1400)<br>1996-2002, 2002-2008<br>Danish National Birth Cohort	Serum	PFOA<br>Q1: <26.1<br>Q2: 26.1-33.3<br>Q3: 33.4-43.2<br>Q4: >43.2	Positive associations with PFOA and hospitalization due to infection in girls and primiparous women, negative associations in boys and multiparous women.	Hospitalization due to infection (IRR)<br><br>PFOA<br>Girls: 1.21 (1.04, 1.42)<br>Multiparous: 0.87 (0.76, 1.00)

			PFOS<br>Q1: <3.91<br>Q2: 3.91-5.20<br>Q3: 5.21-6.96<br>Q4: >6.96	Positive associations with PFOS and hospitalization due to infection in girls and primiparous women, negative associations in boys and multiparous women.	PFOS<br>Girls: 1.18 (1.03, 1.36)<br>Boys: 0.90 (0.80, 1.02)<br>Primiparous: 1.10 (0.96, 1.27)<br>Multiparous: 0.93 (0.83, 1.05)
Okada et al. (2014)	Cohort<br>Japan<br>(n=2063)<br>2003-2009<br>Hokkaido Study on Environment and Children's Health	Serum	PFOA<br>Q1: <1.31<br>Q2: 1.31-2.01<br>Q3: 2.01-3.26<br>Q4: >3.26	Negative associations with higher quartiles of PFOA for allergies and eczema.	PFOA<br>Total allergies<br>Q1: ref<br>Q2: 1.05 (0.81, 1.37)<br>Q3: 0.80 (0.61, 1.06)<br>Q4: 0.79 (0.59, 1.04)
			PFOS<br>Q1: <3.71<br>Q2: 3.71-5.01<br>Q3: 5.02-6.82<br>Q4: >6.82	Potential negative associations with total allergic diseases and eczema with increasing PFOS.	PFOS<br>Total allergic diseases (OR)<br>Q1: ref<br>Q2: 0.97 (0.75, 1.26)<br>Q3: 0.80 (0.61, 1.04)<br>Q4: 0.86 (0.66, 1.13)
Okada et al. (2012)	Cohort<br>Japan<br>(n=268)<br>2003-2005<br>Hokkaido Study on Environment and Children's Health	Serum	1 Log(10)-unit cubic<br>(PFOA: 1.4)	Negative associations with IgE levels and PFOA reported for girls, null associations for boys.	Reported as figures.
			(PFOS: 5.6)	No association between PFOS and cord blood IgE levels.	Reported as figures
Wang et al. (2011)	Cohort<br>Taiwan<br>(n = 239)<br>2004<br>Taiwan Birth Panel Study	Cord blood	PFOA<br>Q1: <0.085<br>Q2: 0.085 - 0.57<br>Q3: 0.57 - 1.085<br>Q4: >1.085	No associations between PFOA levels and atopic dermatitis or serum IgE at 2 years of age. Positive association with cord blood IgE levels in boys.	PFOA<br>Atopic dermatitis (OR)<br>Q1: ref<br>Q2: 0.84 (0.28, 2.48)<br>Q3: 1.03 (0.42, 2.56)<br>Q4: 0.58 (0.22, 1.58)
			PFOS<br>Q1: <1.25<br>Q2: 1.25 –1.835<br>Q3: 1.835-2.775<br>Q4: >2.775	Potential positive associations with highest quartiles of PFOS and atopic dermatitis or serum IgE at 2 years of age. Positive association with cord blood IgE levels in boys.	PFOS<br>Q1: ref<br>Q2: 0.68 (0.20, 2.30)<br>Q3: 2.34 (0.86, 6.41)<br>Q4: 2.19 (0.78, 6.17)
			PFNA<br>Q1: <0.14	Potential positive associations between PFNA and atopic	PFNA<br>Q1: ref

			Q2: 0.140 - 0.765<br>Q3: 0.765 - 2.515<br>Q4: >2.515	dermatitis, though low precision and no trends. No association with serum IgE at 2 years of age.	Q2: 1.46 (0.35, 6.07)<br>Q3: 1.53 (0.59, 3.93)<br>Q4: 0.72 (0.23, 2.21)
Ashley-Martin et al. (2015)	Cohort<br>10 Canadian cities<br>(n = 1258)<br>2008-2011<br>MIREC	Maternal serum	1 log-µg/L<br>(PFOA: 1.7)	No associations between PFOA and newborn immune function markers.	PFOA<br>Elevated (≥80%) IL-33/TSLP (OR)<br>1.1 (0.6, 1.8)<br>Elevated (≥0.5ku/L) IgE (OR)<br>1.1 (0.6, 1.9)
			(PFOS: 4.7)	No associations between PFOS and newborn immune function markers.	PFOS<br>IL-33/TSLP<br>1.1 (0.6, 1.9)<br>IgE<br>1.1 (0.6, 1.9)
			(PFHxS: 1.0)	No associations between PFHxS and newborn immune function markers.	PFHxS<br>Il-33/TSLP<br>1.0 (0.7, 1.4)<br>IgE<br>1.0 (0.7, 1.4)
Granum et al. (2013)	Cohort<br>Norway<br>(n=99, 56)<br>2007-2011<br>MoBa	Maternal plasma	1<br>(PFOA: 1.1)	Associations are unadjusted for potential confounders, positive crude associations found for number of colds and gastroenteritis episodes and PFOA.	PFOA<br>Colds (Δ#)<br>3rd year of life: 0.42 (0.16, 0.72)<br>Gastroenteritis episodes (Δ#)<br>1st-3rd years: 0.31 (0.00, 0.61)
			(PFOS: 5.6)	Associations are unadjusted for potential confounders, no associations found for number of colds and gastroenteritis episodes and PFOS.	PFOS<br>Colds (Δ#)<br>3rd year of life: 0.03 (-0.03, 0.10)<br>Gastroenteritis episodes (Δ#)<br>1st-3rd years: 0.03 (-0.04, 0.10)
			(PFNA: 0.3,<br>PFHxS: 0.3)	Number of colds positively associated with PFNA and PFHxS. Gastroenteritis episodes positively associated only with PFHxS.	PFNA<br>Colds (Δ#)<br>3rd year of life: 1.24 (0.08, 2.40)<br>Gastroenteritis episodes (Δ#)<br>1st-3rd years: -0.10 (-1.36, 1.17)
Vaccination response					
Grandjean et al. (2012)*	Cohort<br>Faroe Islands<br>(n=587)<br>1997-2000, 2008	Serum, maternal and child at 5 years	1 Log(2) unit<br>(PFOA: 3.20)	Negative associations between tetanus and diphtheria antibody levels at 7 years and PFOA concentration at 5 years,	PFOA<br>Tetanus antibodies (%Δ)<br>Age 7, Maternal serum:<br>12.3 (−8.6 to 38.1)

				adjusting for antibody levels at 5 years. No associations with maternal PFOA levels.	Age 7, child serum<br>−35.8 (−51.9 to −14.2)<br>Age 7, child serum & adjusted<br>−28.2 (−42.7 to −10.1)
			(PFOS: 27.3)	Maternal PFOS associated with increase in tetanus antibodies concentration at 5 and 7 years, potential negative association with diphtheria antibodies. Child serum PFOS potentially negatively associated with tetanus and diphtheria antibodies.	PFOS<br>Tetanus antibodies (%Δ)<br>Age 7, Maternal serum:<br>35.3 (−3.9, 90.6)<br>Age 7, child serum<br>-23.8 (-44.3, 4.2)<br>Age 7, child serum & adjusted for age 5<br>−11.4 (−30.5, 12.8)
			(Ranges from 0.28 – 4.41)	Negative associations with increasing PFHxS, PFNA, and PFDA concentrations and tetanus and diphtheria antibody concentrations, primarily with PFASs measured at age 5, though some associations with maternal concentrations as well.	Tetanus antibodies (%Δ)<br>Age 7, child serum<br>PFHxS<br>-19.7 (-31.6, -5.7)<br>PFNA<br>-17.4 (-34.1, 3.6)<br>PFDA<br>-22.3 (-35.8, -5.8)
				Latent factor representing PFOS, PFOA, and PFHxS was created and doubling of concentration at age 5 (pre-booster) was associated with decreased antibody concentrations for tetanus and diphtheria at age 7 (post-booster). Maternal PFAS negatively associated with diphtheria antibodies at both age 5 and age 7, but not tetanus.	Tetanus antibodies, age 5 pre-booster (%Δ)<br>Maternal : -20.2 (-49.2, 25.2)<br>5 year old: -20.5 (-44.4, 13.6)<br>5 year old, adjusted for maternal:<br>-17.2 (-42.1, 18.5)<br><br>Tetanus antibodies, age 7 post-booster (%Δ)<br>Maternal : 35.1 (-25.4, 144.6)<br>5 year old: -55.2 (-73.3, -25)<br>5 year old, adjusted for maternal:<br>-58.8 (-76, -29.3)
Granum et al. (2013)	Cohort<br>Norway<br>(n=99, 56)<br>2007-2011<br>MoBa	Maternal plasma	1<br>(PFOA: 1.1)	Decrease in rubella antibodies associated with increasing PFOA concentration. Potential decrease for measles antibodies, but not adjusted	PFOA<br>Rubella vaccine antibodies (Δ)<br>-0.40 (-0.64, -0.17)<br>Measles vaccine antibodies (Δ)<br>-0.13 (-0.35, 0.09)

				for potential confounders. No association with HiB or tetanus antibodies and PFOA.	
			(PFOS: 5.6)	PFOS negatively associated with rubella and measles antibodies. No association with Hib or tetanus antibodies.	PFOS<br>Rubella vaccine antibodies (Δ)<br>-0.08 (-0.14, -0.02)<br>Measles vaccine antibodies (Δ)<br>-0.05 (-0.10, 0.01)
			(PFNA: 0.3, PFHxS: 0.3)	Rubella antibodies negatively associated with PFNA and PFHxS. Measles Hib and tetanus antibodies not associated with PFNA or PFHxS.	PFNA<br>Rubella vaccine antibodies (Δ)<br>-1.38 (-2.35, -0.40)<br>Measles vaccine antibodies (Δ)<br>-0.55 (-1.51, 0.41)
Stein et al. (2016)	Cross-sectional<br>United States<br>(n = 640)<br>1999-2000, 2003-2004<br>NHANES	serum	1 Log(2)-unit<br>(PFOA: 3.59)	No association between doubling of PFOA and measles antibodies. Decreases in rubella antibodies with PFOA doubling were observed in seropositive individuals. PFOA increases were negatively associated with mumps antibodies.	PFOA<br>Mumps vaccine antibodies (Δ)<br>All: -6.0 (-12.4, 0.9)<br>Seropositive: -6.6 (-11.7, -1.5)<br>Measles vaccine antibodies (Δ)<br>All: -0.1 (-13.8, 15.6)<br>Seropositive: -3.4 (-16.7, 11.9)
			(PFOS: 15.0)	Doubling of PFOS was associated with decreases in mumps and rubella antibodies. No associations with measles antibodies.	PFOS<br>Rubella vaccine antibodies (Δ)<br>All: -8.4 (-17.9, 2.1)<br>Seropositive: -13.3 (-19.9, -6.2)
			(PFNA: 0.929, PFHxS: 2.09)	No association between measles, mumps, or rubella antibodies and PFNA. PFHxS was associated with rubella antibodies in seropositive individuals, but measles and mumps were not.	PFNA<br>Mumps vaccine antibodies (Δ)<br>All: -2.7 (-7.2, 2.0)<br>Seropositive: -2.7 (-8.4, 3.4)<br>PFHxS<br>Rubella vaccine antibodies (Δ)<br>All: -50 (-10.8, 1.2)<br>Seropositive: -6.0 (-9.6, -2.2)

*: Study that examined PFAS as a mixture

Table S5. Studies of perfluorinated compounds and puberty related outcomes.

Study	Study Characteristics	Exposure Matrix	Conc (ng/mL)	Summary of Results	Selected Effect Estimates
Christensen et al. (2011)	Case-control<br>United Kingdom<br>(cases = 218,<br>controls = 230)<br>1991-2004<br>ALSPAC	Maternal serum	1 log-unit<br>(PFOA: 3.7)	No association for menarche onset before age 11.5 with PFOA.	Menarche before age 11.5 (Odds ratio (OR))<br>PFOA: 1.01 (0.61, 1.68)
			(PFOS: 19.8)	Negative association between age at menarche before 11.5 years and PFOS.	PFOS: 0.68 (0.40, 1.13)
			(Ranges from 0.2 to 21.7)	No association with PFNA, PFHxS, PROSA, Et-PFOSA-AcOH, sulfonamide esters, or carboxylates. Potential negative associations for Me-PFOSA-AcOH or sulfonates.	PFNA: 0.91 (0.59, 1.4)<br>Sulfonates: 0.66 (0.4, 1.08)
Kristensen et al. (2013)	Cohort<br>Denmark<br>(n = 343)<br>1988-2008<br>Danish Pregnancy Cohort	Maternal serum at gestational week 30	PFOA<br>T1: <3.0<br>T2: 3.0 – 4.3<br>T3: >4.3	PFOA associated with later age at menarche, no associations with other reproductive parameters examined.	Age at menarche (Δ months) per tertile increase<br>PFOA: 1.01 (0.22, 1.89)
			PFOS<br>T1: <18.0<br>T2: 18.0 – 23.6<br>T3: >23.6	No association with age at menarche, menstrual cycle length, follicle number or reproductive hormone concentrations and PFOS.	Age at menarche (Δ months) per tertile increase<br>PFOS: 0.12 (-0.10, 0.34)<br>Cycle length (Δ days), nonusers of hormonal birth control per tertile increase<br>PFOS: 0.001 (-0.007, 0.008)
Lopez-Espinosa et al. (2011)	Cross-sectional<br>United States<br>(n = 3076 boys, 2931 girls)<br>2005-2006<br>C8	Serum	1 ln-unit<br>(PFOA: 23)	Later age at menarche and higher concentrations of pubertal indicators associated with PFOA in girls. No associations with pubertal indicators and PFOA in boys.	Menarche status (OR)<br>PFOA: 0.83 (0.73, 0.95)<br>PFOA, adjusted for PFOS: 0.87 (0.75, 1.00)
			(PFOS: 19.4)	Negative associations for hormone concentrations (testosterone and estradiol) and menarche with PFOS.	PFOS: 0.49 (0.36, 0.67)<br>PFOS, adjusted for PFOA: 0.67 (0.48, 0.93)
Maisonet et al. (2015a)	Cohort, subset<br>Avon, UK	Maternal serum	Tertiles<br>PFOA	Higher tertiles of PFOA associated with higher levels of	Total testosterone (Δ nmol/L)

	(n = 72 girls)<br>1991-1992, recruitment<br>ALSPAC		T1: <2.9<br>T2: 2.9-4.1<br>T3: >4.1	total testosterone in girls. No association with serum sex hormone-binding globulin concentrations.	PFOA<br>T1: ref<br>T2: 0.15 (-0.02, 0.32)<br>T3: 0.24 (0.05, 0.43)
			PFOS<br>T1: <15.9<br>T2: 15.9-22.6<br>T3: >22.6	Higher tertiles of PFOS associated with higher levels of total testosterone in girls. No association with serum sex hormone-binding globulin concentrations.	PFOS<br>T1: ref<br>T2: 0.1 (-0.07, 0.28)<br>T3: 0.18 (0.01, 0.35)
			PFHxS<br>T1: <1.3<br>T2: 1.3-1.9<br>T3: >1.9<br><br>PFNA<br>T1: <0.5<br>T2: 0.5-0.6<br>T3: >0.6	PFHxS associated with increased testosterone, no association with sex hormone-binding globulin. PFNA not associated with either.	PFHxS<br>T1: ref<br>T2: 0.18 (0.00, 0.37)<br>T3: 0.18 (0.00, 0.35)<br><br>PFNA<br>T1: ref<br>T2: 0.08 (-0.10, 0.25)<br>T3: 0.05 (-0.14, 0.24)
Lopez-Espinosa et al. (2016)	Cross-sectional<br>United States<br>(n = 2292)<br>2005-2006<br>C8	Serum	IQR (median)<br><br>PFOA<br>Boys: 1.68 (34.8)<br>Girls: 1.70 (30.1)	PFOA concentrations were associated with decreased (log transformed) levels of insulin-like growth factor-1 (IGF-1) in girls. In both sexes, PFOA was potentially associated with decreased total testosterone and increased ln-estradiol.	IGF-1 (% difference)<br>PFOA<br>Boys: -0.4 (-3.4, 2.7)<br>Girls: -3.6 (-6.6, -0.5)
			PFOS<br>Boys: 0.66 (22.4)<br>Girls: 0.65 (20.9)	PFOS was negatively associated with total testosterone, and IGF-1. In boys only PFOS was negatively associated with estradiol.	PFOS<br>Boys: -5.9 (-8.3, -3.3)<br>Girls: -5.6 (-8.2, -2.9)
			PFNA<br>Boys: 0.57 (1.7)<br>Girls: 0.61 (1.7)<br><br>PFHxS<br>Boys: 1.40 (8.1)<br>Girls: 1.29 (7.0)	PFNA was negatively associated with IGF-1.<br>There was no evidence of associations with PFHxS and sex hormones or IGF-1	PFNA<br>Boys: -3.5 (-6.0, -1.0)<br>Girls: -3.8 (-6.4, -1.2)
Tsai et al. (2015)	Cross-sectional<br>Taiwan<br>(n = 540)<br>2006-2008	Serum	(PFOA: 2.74)	Increasing PFOA percentile categories were not associated with log transformed follicle stimulating hormone (FSH)	Effects are reported as mean levels and p-value for trend only.

				concentrations among 12-17 year olds.	
			(PFOS: 7.78)	PFOS was associated with decreased FSH in 12-17 year old males.	
			(PFNA: 1.10 PFUA: 5.84)	PFNA was not associated with FSH, while PFUA was negatively associated with FSH in 12-17 year old females.	

Table S6. Studies of perfluorinated compounds and thyroid related outcomes.

Study	Study Characteristics	Exposure matrix	Conc ng/mL	Summary of results	Selected effect estimates
Lopez-Espinosa et al. (2012)	Cross-sectional<br>United States<br>(n = 4713)<br>2005-2006<br>C8	Serum	IQR increase<br><br>PFOA<br>Q1: <13.1<br>Q2: 13.1 - 29.2<br>Q3: 29.3 – 67.6<br>Q4: >67.6	Hyperthyroidism associated with PFOA.<br>No associations between thyroid hormone levels and PFOA.	Hypothyroidism (OR)<br>PFOA: 1.54 (1.00, 2.37)<br>TSH (log- μIU/mL)<br>PFOA:<br>Q1: ref<br>Q2: 1.0 (–1.9, 4.0)<br>Q3: 1.0 (–2.0, 4.1)<br>Q4: 2.4 (–0.6, 5.5)
			PFOS<br>Q1: <14.5<br>Q2: 14.5-19.9<br>Q3: 20.0-27.7<br>Q4: >27.7	Increases in TSH and TT4 at highest PFOS concentrations only.	Hypothyroidism (OR)<br>PFOS: 0.91 (0.63, 1.31)<br>TSH (log- μIU/mL)<br>PFOS:<br>Q1: ref<br>Q2: 0.3 (-2.6, 3.2)<br>Q3: -1.3 (-4.2, 1.7)<br>Q4: 3.1 (0, 6.2)
			PFNA:<br>Q1: <1.2<br>Q2: 1.2-1.4<br>Q3: 1.5-1.9<br>Q4: >1.9	No associations between TSH levels and PFNA. TT4 levels show increases with increasing PFNA.	Hypothyroidism (OR)<br>PFNA: 1.11 (0.77, 1.60)<br>TSH (log- μIU/mL)<br>PFNA:<br>Q1: ref<br>Q2: 0.4 (-2.6, 3.5)<br>Q3: -0.3 (-3.2, 2.6)<br>Q4: 1.5 (-1.6, 4.6)
Lin et al. (2013b)	Cohort<br>Taiwan	Serum	1<br>(PFOA: 3.64)	No association between levels of free T4 and log-TSH and PFOA	Free T4 (ng/dL)<br>PFOA: 0.001 (-0.002, 0.003)

	(n = 531)<br>1999-2000<br>Young Taiwanese<br>Cardiovascular Cohort Study				Log-TSH (m IU/L)<br>PFOA: -0.006 (-0.019, 0.008)
			(PFOS: 5.47)	No association between levels of free T4 and log-TSH and PFOS	Free T4 (ng/dL)<br>PFOS: 0.001 (0.000-0.002)<br>Log-TSH (m IU/L)<br>PFOS: 0.006 (-0.013-0.025)
			(PFUA: 1.50<br>PFNA: 1.2)	No association between levels of free T4 and log-TSH and PFUA. PFNA has small positive association with free T4 but no association with TSH. Some potential positive ORs for hypothyroidism with both PFNA and PFUA, however case numbers are small and confidence intervals very wide.	Free T4 (ng/dL)<br>PFNA: 0.004 (0.001-0.007)<br>Log-TSH (m IU/L)<br>PFNA: -0.006 (-0.019, 0.008)<br><br>Free T4 (ng/dL)<br>PFUA: 0.000(0.000-0.001)<br>Log-TSH (m IU/L)<br>PFUA: 0.001 (-0.004-0.007)
de Cock et al. (2014)	Cohort<br>The Netherlands<br>(n = 83)<br>2011-2013	Cord blood	PFOA<br>Q1: <0.059<br>Q2: 0.059 – 0.865<br>Q3: 0.865 – 1.20<br>Q4: >1.20	Potential positive association between PFOA and T4 levels in girls, but sample size and exposure variability are small.	T4 levels (Δ nmol/L)<br>PFOA, boys<br>Q1: ref<br>Q2: 7.9 (-18.04, 33.92)<br>Q3: -2.1 (-20.94, 16.78)<br>Q4: 6.2 (-16.08, 28.5)<br>PFOA, girls<br>Q1: ref<br>Q2: -5.9 (-26.75, 14.94)<br>Q3: 11.8 (-19.08, 42.72)<br>Q4: 38.6 (13.34, 63.83)
			PFOS<br>Q1: <1.03<br>Q2: 1.03 – 1.60<br>Q3: 1.60 – 2.18<br>Q4: 2.18	No associations between PFOS and T4 levels.	PFOS, boys<br>Q1: ref<br>Q2: -7.9 (-31.56, 15.74)<br>Q3: -16.5 (-40.32, 7.34)<br>Q4: -9.6 (-32.57, 13.31)<br>PFOS, girls<br>Q1: ref<br>Q2: -1.3 (-30.45, 27.94)<br>Q3: 4.5 (-25.95, 34.92)<br>Q4: 15.9 (-10.67, 42.4)
Kim et al. (2011)	Cross-sectional<br>South Korea<br>(n = 44)	Cord blood	Median<br>PFOA: 1.46	Small negative correlations between PFOA and thyroid hormones from cord blood.	Adjusted Pearson correlation<br>Maternal PFOA:<br>Fetal T3: -0.240

	8/2008 – 3/2009				Fetal T4: -0.157<br>Fetal TSH: 0.089
			PFOS: 2.93	Negative correlations between maternal PFOS and fetal T3 positive correlation with fetal TSH.	Maternal PFOS<br>Fetal T3: -0.414<br>Fetal T4: -0.071<br>Fetal TSH: 0.443
			PFNA: 0.44<br>PFDA: 0.31<br>PFHxS: 0.55<br>PFHpS: 0.09<br>PFUnDA: 0.60<br>PFTrDA: 0.24	Maternal PFTrDA negatively correlated with fetal T3 and T4. PFHxS small negative correlation with T3.	Maternal PFHxS<br>Fetal T3: -0.261<br>Fetal T4: 0.030<br>Fetal TSH: 0.091<br>Maternal PFTrDA<br>Fetal T3: -0.380<br>Fetal T4: -0.441<br>Fetal TSH: 0.288
Kim et al. (2016)	Cross-sectional<br>South Korea<br>(n = 27 cases, 13 controls)<br>July 2009- Feb 2010	Serum	(PFOA: 2.12, 5.398)<br>(PFOS: 4.05, 5.326)	Mean levels of PFOA, PFNA, PFDA, PFUnDA, and total PFASs were higher in infants with congenital hypothyroidism than in health infants.	Reported as figures.
Kato et al. (2016)	Cross-sectional<br>Japan<br>(n = 392 mother-infant pairs)<br>2002 – 2005<br>Hokkaido Study on the Environment and Children's Health	Maternal serum	log(10)-unit<br>(PFOA: 1.2)	PFOA was not associated with infant levels of thyroid stimulating hormone (TSH) or free thyroxine (FT4).	PFOA<br>FT4 (Δ log(10) ng/mL): 0.003 ($p = 0.960$)<br>TSH (Δ log(10) lU/mL): -0.014 ($p = 0.801$)
			(PFOS: 5.2)	Log(10) increases in PFOS were associated with increases in TSH, but not FT4.	PFOS<br>FT4 (Δ log(10) ng/mL): -0.043 ($p = 0.452$)<br>TSH (Δ log(10) lU/mL): 0.177 ($p = 0.001$)
Tsai et al. (2017)	Cross-sectional<br>Taiwan<br>(n = 118 mother-infant pairs)<br>April 2004 – January 2005<br>Taiwan Birth Panel Study	Cord blood plasma	1 Log-unit<br>(PFOA: 3.14)	No association between concentration of PFOA and T4, TSH, or T3 in either boys or girls.	PFOA<br>T4 (Δ log µg/dL), boys<br>-0.082 (-0.643, 0.48)
			(PFOS: 7.24)	Increasing log-PFOS associated with decreasing log-T4 in boys, but not in girls. Increasing log-PFOS also associated with increasing log-TSH in both boys and girls, but percentile analyses	PFOS<br>T4 (Δ log µg/dL), boys<br>-0.667 (-1.283, -0.05)<br>>90th percentile v. <30th: -2.115 (-3.622, -0.608)

				show non-linear response and stronger magnitude of effects in boys. PFOS not associated with T3 in boys, potential positive association in girls.	T4 (Δ log µg/dL), girls<br>0.033 (-0.707, 0.773)<br>$>90^{th}$ percentile v. $<30^{th}$: 0.808 (-0.954, 2.571)<br><br>TSH (Δ log µIU/mL),<br>Boys: 0.333 (0.012, 0.678)<br>Girls: 0.36 (-0.033, 0.753)
			(PFUnDA: 15.94)<br>(PFNA: 7.55)	No association between concentration of PFUnDA and T4 or T3 in either boys or girls. Potential positive association with TSH in boys only. No association between concentration of PFNA and T4, TSH, or T3 in either boys or girls.	PFUnDA<br>T4 (Δ log µg/dL)<br>Boys: -0.082 (-0.485, 0.321)<br><br>TSH (Δ log µIU/mL)<br>Boys: 0.142 (-0.062, 0.346)

Table S7. Studies of perfluorinated compounds and renal related outcomes.

Study	Study Characteristics	Exposure matrix	Conc ng/mL	Summary of results	Selected effect estimates
Watkins et al. (2013)	Cross-sectional<br>United States<br>(n = 9660)<br>2005-2006<br>C8	Serum	PFOA: 1.63	Increases in PFOA associated with decreased eGFR.	eGFR (Δ rate)<br><br>PFOA<br>-0.73 (-1.38, -0.08)
			PFOS: 0.64	Increases in PFOS associated with decreased eGFR.	PFOS<br>-1.34 (-1.91, -0.77)
			PFNA: 0.51<br>PFHxS: 1.27<br>Ln-unit	Increases in log-concentrations of PFASs negatively associated with eGRF.	PFNA<br>-0.88 (-1.41, -0.36)<br><br>PFHxS<br>-1.02 (-1.64, -0.40)
Lin et al. (2013b)	Cohort<br>Taiwan<br>(n = 531)<br>1999-2000<br>Young Taiwanese Cardiovascular Cohort Study	Serum	(PFOA: 3.64)<br>(PFOS: 5.47)<br>(PFUA: 1.50)<br>(PFNA: 1.2)	PFUA levels higher in children with chronic renal failure.	PFUA, ng/mL geometric mean (SD)<br>No renal failure: 5.68 (2.92)<br>Chronic renal failure: 11.07 (2.64)

Kataria et al. (2015)	Cross-sectional<br>United States<br>(n = 1960)<br>2003-2010<br>NHANES	Serum	PFOA<br>Q1: <2.5 ng/mL<br>Q2: 2.5-3.5<br>Q3: 3.5-4.7<br>Q4: >=4.7	Increases in PFOA were negatively associated with eGFR and positively associated with uric acid concentrations, this held true after adjusting for PFOS as well. No associations with creatinine.	eGFR (Δ rate)<br><br>PFOA<br>Q1: ref<br>Q2: −2.81 (−7.16, 1.53)<br>Q3: −5.50 (−11.50, 0.50)<br>Q4: −6.84 (−11.48, −2.19)
			PFOS<br>Q1: <7.9<br>Q2: 7.9-12.8<br>Q3: 12.8-19.4<br>Q4: >=19.4	Increases in PFOS were negatively associated with eGFR and positively associated with uric acid concentrations. This was attenuated after adjustment for PFOA.	PFOS<br>Q1: ref<br>Q2: −5.39 (−9.87, −0.92)<br>Q3: −7.25 (−12.25, −2.25)<br>Q4: −9.69 (−14.78, −4.59)
			PFNA<br>Q1: <0.7<br>Q2: 0.7-1.0<br>Q3: 1.0-1.5<br>Q4: >=1.5	PFNA and PFHxS were not associated with , eGFR, creatinine, or uric acid concentration.	PFNA<br>Q1: ref<br>Q2: 1.25 (−4.30, 6.80)<br>Q3: 2.59 (−1.89, 7.08)<br>Q4: −1.2 (−5.62, 3.22)
Qin et al. (2016)	Cross-sectional<br>Taiwan<br>(n = 225 non-asthmatics)<br>2009-2010<br>Genetics and Biomarkers study for Childhood Asthma (GBCA)	Serum	Quartile increase<br>(PFOA: 0.5)	Increasing PFOA was associated with increased odds of high uric acid level (≥6 mg/dL). Potentially higher odds in boys than girls.	High uric acid level (OR)<br><br>PFOA<br>2.16 (1.29, 3.61)
			(PFOS: 28.9)	PFOS was potentially associated with high uric acid level.	PFOS<br>1.35 (0.95, 1.93)
			(Ranges from 0.2 to 5.0)	PFBS, PFDA, PFHxS, and PFNA were potentially associated with high uric acid level. PFDOA, PFHxA, and PFTA were not associated with high uric acid level.	PFNA<br>1.28 (0.83, 1.96)<br><br>PFTA<br>0.97 (0.69, 1.36)

References

Andersen CS, Fei C, Gamborg M, Nohr EA, Sorensen TI, Olsen J. 2010. Prenatal exposures to perfluorinated chemicals and anthropometric measures in infancy. American journal of epidemiology 172:1230-1237.

Andersen CS, Fei C, Gamborg M, Nohr EA, Sorensen TI, Olsen J. 2013. Prenatal exposures to perfluorinated chemicals and anthropometry at 7 years of age. American journal of epidemiology 178:921-927.

Ashley-Martin J, Dodds L, Levy AR, Platt RW, Marshall JS, Arbuckle TE. 2015. Prenatal exposure to phthalates, bisphenol a and perfluoroalkyl substances and cord blood levels of ige, tslp and il-33. Environmental research 140:360-368.

Braun JM, Chen A, Romano ME, Calafat AM, Webster GM, Yolton K, et al. 2016. Prenatal perfluoroalkyl substance exposure and child adiposity at 8 years of age: The home study. Obesity 24:231-237.

Chen MH, Ha EH, Liao HF, Jeng SF, Su YN, Wen TW, et al. 2013. Perfluorinated compound levels in cord blood and neurodevelopment at 2 years of age. Epidemiology (Cambridge, Mass) 24:800-808.

Christensen KY, Maisonet M, Rubin C, Holmes A, Calafat AM, Kato K, et al. 2011. Exposure to polyfluoroalkyl chemicals during pregnancy is not associated with offspring age at menarche in a contemporary british cohort. Environment international 37:129-135.

de Cock M, de Boer MR, Lamoree M, Legler J, van de Bor M. 2014. Prenatal exposure to endocrine disrupting chemicals in relation to thyroid hormone levels in infants - a dutch prospective cohort study. Environmental health : a global access science source 13:106.

Donauer S, Chen A, Xu Y, Calafat AM, Sjodin A, Yolton K. 2015. Prenatal exposure to polybrominated diphenyl ethers and polyfluoroalkyl chemicals and infant neurobehavior. The Journal of pediatrics 166:736-742.

Dong GH, Tung KY, Tsai CH, Liu MM, Wang D, Liu W, et al. 2013. Serum polyfluoroalkyl concentrations, asthma outcomes, and immunological markers in a case-control study of taiwanese children. Environmental health perspectives 121:507-513, 513e501-508.

Fei C, McLaughlin JK, Lipworth L, Olsen J. 2008. Prenatal exposure to perfluorooctanoate (pfoa) and perfluorooctanesulfonate (pfos) and maternally reported developmental milestones in infancy. Environmental health perspectives 116:1391-1395.

Fei C, McLaughlin JK, Lipworth L, Olsen J. 2010. Prenatal exposure to pfoa and pfos and risk of hospitalization for infectious diseases in early childhood. Environmental research 110:773-777.

Fei C, Olsen J. 2011. Prenatal exposure to perfluorinated chemicals and behavioral or coordination problems at age 7 years. Environmental health perspectives 119:573-578.

Forns J, Iszatt N, White RA, Mandal S, Sabaredzovic A, Lamoree M, et al. 2015. Perfluoroalkyl substances measured in breast milk and child neuropsychological development in a norwegian birth cohort study. Environment international 83:176-182.

Frisbee SJ, Shankar A, Knox SS, Steenland K, Savitz DA, Fletcher T, et al. 2010. Perfluorooctanoic acid, perfluorooctanesulfonate, and serum lipids in children and adolescents: Results from the c8 health project. Archives of pediatrics & adolescent medicine 164:860-869.

Geiger SD, Xiao J, Ducatman A, Frisbee S, Innes K, Shankar A. 2014a. The association between pfoa, pfos and serum lipid levels in adolescents. Chemosphere 98:78-83.

Geiger SD, Xiao J, Shankar A. 2014b. No association between perfluoroalkyl chemicals and hypertension in children. Integrated blood pressure control 7:1.

Goudarzi H, Nakajima S, Ikeno T, Sasaki S, Kobayashi S, Miyashita C, et al. 2016. Prenatal exposure to perfluorinated chemicals and neurodevelopment in early infancy: The hokkaido study. The Science of the total environment 541:1002-1010.

Grandjean P, Andersen EW, Budtz-Jorgensen E, Nielsen F, Molbak K, Weihe P, et al. 2012. Serum vaccine antibody concentrations in children exposed to perfluorinated compounds. Jama 307:391-397.

Granum B, Haug LS, Namork E, Stolevik SB, Thomsen C, Aaberge IS, et al. 2013. Pre-natal exposure to perfluoroalkyl substances may be associated with altered vaccine antibody levels and immune-related health outcomes in early childhood. Journal of immunotoxicology 10:373-379.

Gump BB, Wu Q, Dumas AK, Kannan K. 2011. Perfluorochemical (pfc) exposure in children: Associations with impaired response inhibition. Environmental science & technology 45:8151-8159.

Halldorsson TI, Rytter D, Haug LS, Bech BH, Danielsen I, Becher G, et al. 2012. Prenatal exposure to perfluorooctanoate and risk of overweight at 20 years of age: A prospective cohort study. Environmental health perspectives 120:668-673.

Hoffman K, Webster TF, Weisskopf MG, Weinberg J, Vieira VM. 2010. Exposure to polyfluoroalkyl chemicals and attention deficit/hyperactivity disorder in u.S. Children 12-15 years of age. Environmental health perspectives 118:1762-1767.

Hoyer BB, Ramlau-Hansen CH, Obel C, Pedersen HS, Hernik A, Ogniev V, et al. 2015a. Pregnancy serum concentrations of perfluorinated alkyl substances and offspring behaviour and motor development at age 5-9 years--a prospective study. Environmental health : a global access science source 14:2.

Hoyer BB, Ramlau-Hansen CH, Vrijheid M, Valvi D, Pedersen HS, Zviezdai V, et al. 2015b. Anthropometry in 5- to 9-year-old greenlandic and ukrainian children in relation to prenatal exposure to perfluorinated alkyl substances. Environmental health perspectives 123:841-846.

Humblet O, Diaz-Ramirez LG, Balmes JR, Pinney SM, Hiatt RA. 2014. Perfluoroalkyl chemicals and asthma among children 12-19 years of age: Nhanes (1999-2008). Environmental health perspectives 122:1129-1133.

Kataria A, Trachtman H, Malaga-Dieguez L, Trasande L. 2015. Association between perfluoroalkyl acids and kidney function in a cross-sectional study of adolescents. Environmental health : a global access science source 14:89.

Kato S, Itoh S, Yuasa M, Baba T, Miyashita C, Sasaki S, et al. 2016. Association of perfluorinated chemical exposure in utero with maternal and infant thyroid hormone levels in the sapporo cohort of hokkaido study on the environment and children's health. Environmental health and preventive medicine.

Kim DH, Kim UJ, Kim HY, Choi SD, Oh JE. 2016. Perfluoroalkyl substances in serum from south korean infants with congenital hypothyroidism and healthy infants - its relationship with thyroid hormones. Environmental research 147:399-404.

Kim S, Choi K, Ji K, Seo J, Kho Y, Park J, et al. 2011. Trans-placental transfer of thirteen perfluorinated compounds and relations with fetal thyroid hormones. Environmental science & technology 45:7465-7472.

Kristensen SL, Ramlau-Hansen CH, Ernst E, Olsen SF, Bonde JP, Vested A, et al. 2013. Long-term effects of prenatal exposure to perfluoroalkyl substances on female reproduction. Human reproduction (Oxford, England) 28:3337-3348.

Lien GW, Huang CC, Shiu JS, Chen MH, Hsieh WS, Guo YL, et al. 2016. Perfluoroalkyl substances in cord blood and attention deficit/hyperactivity disorder symptoms in seven-year-old children. Chemosphere 156:118-127.

Liew Z, Ritz B, von Ehrenstein OS, Bech BH, Nohr EA, Fei C, et al. 2015. Attention deficit/hyperactivity disorder and childhood autism in association with prenatal exposure to perfluoroalkyl substances: A nested case-control study in the danish national birth cohort. Environmental health perspectives 123:367-373.

Lin CY, Chen PC, Lin YC, Lin LY. 2009. Association among serum perfluoroalkyl chemicals, glucose homeostasis, and metabolic syndrome in adolescents and adults. Diabetes care 32:702-707.

Lin CY, Wen LL, Lin LY, Wen TW, Lien GW, Chen CY, et al. 2011. Associations between levels of serum perfluorinated chemicals and adiponectin in a young hypertension cohort in taiwan. Environmental science & technology 45:10691-10698.

Lin CY, Lin LY, Wen TW, Lien GW, Chien KL, Hsu SH, et al. 2013a. Association between levels of serum perfluorooctane sulfate and carotid artery intima-media thickness in adolescents and young adults. International journal of cardiology 168:3309-3316.

Lin CY, Wen LL, Lin LY, Wen TW, Lien GW, Hsu SH, et al. 2013b. The associations between serum perfluorinated chemicals and thyroid function in adolescents and young adults. Journal of hazardous materials 244-245:637-644.

Lopez-Espinosa MJ, Fletcher T, Armstrong B, Genser B, Dhatariya K, Mondal D, et al. 2011. Association of perfluorooctanoic acid (pfoa) and perfluorooctane sulfonate (pfos) with age of puberty among children living near a chemical plant. Environmental science & technology 45:8160-8166.

Lopez-Espinosa MJ, Mondal D, Armstrong B, Bloom MS, Fletcher T. 2012. Thyroid function and perfluoroalkyl acids in children living near a chemical plant. Environmental health perspectives 120:1036-1041.

Lopez-Espinosa MJ, Mondal D, Armstrong BG, Eskenazi B, Fletcher T. 2016. Perfluoroalkyl substances, sex hormones, and insulin-like growth factor-1 at 6-9 years of age: A cross-sectional analysis within the c8 health project. Environmental health perspectives.

Maisonet M, Terrell ML, McGeehin MA, Christensen KY, Holmes A, Calafat AM, et al. 2012. Maternal concentrations of polyfluoroalkyl compounds during pregnancy and fetal and postnatal growth in british girls. Environmental health perspectives 120:1432-1437.

Maisonet M, Calafat AM, Marcus M, Jaakkola JJ, Lashen H. 2015a. Prenatal exposure to perfluoroalkyl acids and serum testosterone concentrations at 15 years of age in female alspac study participants. Environmental health perspectives.

Maisonet M, Nayha S, Lawlor DA, Marcus M. 2015b. Prenatal exposures to perfluoroalkyl acids and serum lipids at ages 7 and 15 in females. Environment international 82:49-60.

Ode A, Kallen K, Gustafsson P, Rylander L, Jonsson BA, Olofsson P, et al. 2014. Fetal exposure to perfluorinated compounds and attention deficit hyperactivity disorder in childhood. PloS one 9:e95891.

Okada E, Sasaki S, Saijo Y, Washino N, Miyashita C, Kobayashi S, et al. 2012. Prenatal exposure to perfluorinated chemicals and relationship with allergies and infectious diseases in infants. Environmental research 112:118-125.

Okada E, Sasaki S, Kashino I, Matsuura H, Miyashita C, Kobayashi S, et al. 2014. Prenatal exposure to perfluoroalkyl acids and allergic diseases in early childhood. Environment international 65:127-134.

Qin XD, Qian Z, Vaughn MG, Huang J, Ward P, Zeng XW, et al. 2016. Positive associations of serum perfluoroalkyl substances with uric acid and hyperuricemia in children from taiwan. Environmental pollution 212:519-524.

Qin XD, Qian ZM, Dharmage SC, Perret J, Geiger SD, Rigdon SE, et al. 2017. Association of perfluoroalkyl substances exposure with impaired lung function in children. Environmental research 155:15-21.

Quaak I, de Cock M, de Boer M, Lamoree M, Leonards P, van de Bor M. 2016. Prenatal exposure to perfluoroalkyl substances and behavioral development in children. Int J Environ Res Public Health 13.

Smit LA, Lenters V, Hoyer BB, Lindh CH, Pedersen HS, Liermontova I, et al. 2015. Prenatal exposure to environmental chemical contaminants and asthma and eczema in school-age children. Allergy 70:653-660.

Stein CR, Savitz DA. 2011. Serum perfluorinated compound concentration and attention deficit/hyperactivity disorder in children 5-18 years of age. Environmental health perspectives 119:1466-1471.

Stein CR, Savitz DA, Bellinger DC. 2013. Perfluorooctanoate and neuropsychological outcomes in children. Epidemiology (Cambridge, Mass) 24:590-599.

Stein CR, Savitz DA, Bellinger DC. 2014. Perfluorooctanoate exposure in a highly exposed community and parent and teacher reports of behaviour in 6-12-year-old children. Paediatric and perinatal epidemiology 28:146-156.

Stein CR, McGovern KJ, Pajak AM, Maglione PJ, Wolff MS. 2016. Perfluoroalkyl and polyfluoroalkyl substances and indicators of immune function in children aged 12-19 y: National health and nutrition examination survey. Pediatric research 79:348-357.

Strom M, Hansen S, Olsen SF, Haug LS, Rantakokko P, Kiviranta H, et al. 2014. Persistent organic pollutants measured in maternal serum and offspring neurodevelopmental outcomes--a prospective study with long-term follow-up. Environment international 68:41-48.

Timmermann CA, Rossing LI, Grontved A, Ried-Larsen M, Dalgard C, Andersen LB, et al. 2014. Adiposity and glycemic control in children exposed to perfluorinated compounds. The Journal of clinical endocrinology and metabolism 99:E608-614.

Tsai MS, Lin CY, Lin CC, Chen MH, Hsu SH, Chien KL, et al. 2015. Association between perfluoroalkyl substances and reproductive hormones in adolescents and young adults. International journal of hygiene and environmental health 218:437-443.

Tsai MS, Lin CC, Chen MH, Hsieh WS, Chen PC. 2017. Perfluoroalkyl substances and thyroid hormones in cord blood. Environmental pollution 222:543-548.

Vuong AM, Yolton K, Webster GM, Sjodin A, Calafat AM, Braun JM, et al. 2016. Prenatal polybrominated diphenyl ether and perfluoroalkyl substance exposures and executive function in school-age children. Environmental research 147:556-564.

Wang IJ, Hsieh WS, Chen CY, Fletcher T, Lien GW, Chiang HL, et al. 2011. The effect of prenatal perfluorinated chemicals exposures on pediatric atopy. Environmental research 111:785-791.

Wang Y, Rogan WJ, Chen HY, Chen PC, Su PH, Chen HY, et al. 2015. Prenatal exposure to perfluroalkyl substances and children's iq: The taiwan maternal and infant cohort study. International journal of hygiene and environmental health.

Wang Y, Adgent M, Su PH, Chen HY, Chen PC, Hsiung CA, et al. 2016. Prenatal exposure to perfluorocarboxylic acids (pfcas) and fetal and postnatal growth in the taiwan maternal and infant cohort study. Environmental health perspectives.

Watkins DJ, Josson J, Elston B, Bartell SM, Shin HM, Vieira VM, et al. 2013. Exposure to perfluoroalkyl acids and markers of kidney function among children and adolescents living near a chemical plant. Environmental health perspectives 121:625-630.

Zeng XW, Qian Z, Emo B, Vaughn M, Bao J, Qin XD, et al. 2015. Association of polyfluoroalkyl chemical exposure with serum lipids in children. The Science of the total environment 512-513:364-370.

Zhu Y, Qin XD, Zeng XW, Paul G, Morawska L, Su MW, et al. 2016. Associations of serum perfluoroalkyl acid levels with t-helper cell-specific cytokines in children: By gender and asthma status. The Science of the total environment 559:166-173.
